# Cold Atmospheric Plasma Jet Promotes Wound Healing Through CK2-Coordinated PI3K/AKT and MAPK Signaling Pathways

**DOI:** 10.1016/j.mcpro.2025.100962

**Published:** 2025-04-03

**Authors:** Pei-Shan Wu, Tzu-Hsuan Wong, Chun-Wei Hou, Teng-Ping Chu, Jyh-Wei Lee, Bih-Show Lou, Miao-Hsia Lin

**Affiliations:** 1 Chemistry Division, Center for General Education, Chang Gung University, Taoyuan, Taiwan; 2 Department of Microbiology, National Taiwan University College of Medicine, Taipei, Taiwan; 3 Center for Plasma and Thin Film Technologies, Ming Chi University of Technology, New Taipei, Taiwan; 4 International PhD. Program in Plasma and Thin Film Technology, Ming Chi University of Technology, New Taipei, Taiwan; 5 Department of Materials Engineering, Ming Chi University of Technology, New Taipei, Taiwan; 6 High Entropy Materials Center, National Tsing Hua University, Hsinchu, Taiwan; 7 College of Engineering, Chang Gung University, Taoyuan, Taiwan; 8 Department of Orthopaedic Surgery, New Taipei Municipal TuCheng Hospital, Chang Gung Memorial Hospital, Taoyuan, Taiwan

**Keywords:** CK2 kinase, cold atmospheric plasma jet (CAPJ), PI3K/AKT and MAPK signaling, plasma activated medium (PAM), wound healing

## Abstract

The promising role of cold atmospheric plasma jet (CAPJ) treatment in promoting wound healing has been widely documented in therapeutic implications. However, the fact that not all subjects respond equally to CAPJ necessitates the investigation of the underlying cellular mechanisms, which have been rarely understood so far. Given that wound healing is a complex and prolonged process, post plasma-activated medium (PAM) treated keratinocytes were collected at two time points, 2 h (receiving) and 24 h (recovery), for (phospho)proteomic analysis to systematically dissect the molecular basis of CAPJ-promoted wound healing. The receiving (phospho)proteomics datasets, referred to the time point of 2 h, revealed an apparent increase in the phosphorylation of CK2 and its-mediated PI3K/AKT and MAPK signaling pathways, accompanied by a prompted downstream physiological response of cell migration. Additionally, incorporating the network analysis of predicted kinases and their direct interactors, we reiterated that CAPJ influenced cell growth and migration, thereby paving the way for its role in subsequent wound healing processes. Further determining the proteome profiles at recovery phase, which is the time point of 24 h, displayed a totally different view from the receiving proteome which had almost no change. The upregulation of ROBOs/SLITs expression and vesicle trafficking and fusion-related proteins, along with the abundant presence of 14-3-3 family proteins, indicated that the persistent effect of PAM on the wound healing process could potentially promote keratinocyte-fibroblast cross talk and stimulate extracellular matrix synthesis upon epithelialization. Consistent with proteome patterns, CAPJ-treated wound tissues indeed showed a denser and well-organized extracellular matrix architecture, implying hastened epithelialization during wound healing. Collectively, we delineated the molecular basis of CAPJ-accelerated wound healing at early and late responses, providing valuable insights for treatment selection and the development of therapeutic strategies to achieve better outcomes.

## Highlights

• Protein phosphorylation acts as the immediate responders to CAPJ treatment.
• CAPJ-activated PI3K/AKT and MAPK signaling directs keratinocyte migration.
• CK2-coordinated PI3K/AKT and MAPK pathways are key to CAPJ-enhanced wound closure.
• Persistent CAPJ effects enhanced ECM remodeling during wound epithelialization.

## Graphical Abstract

**Figure:**
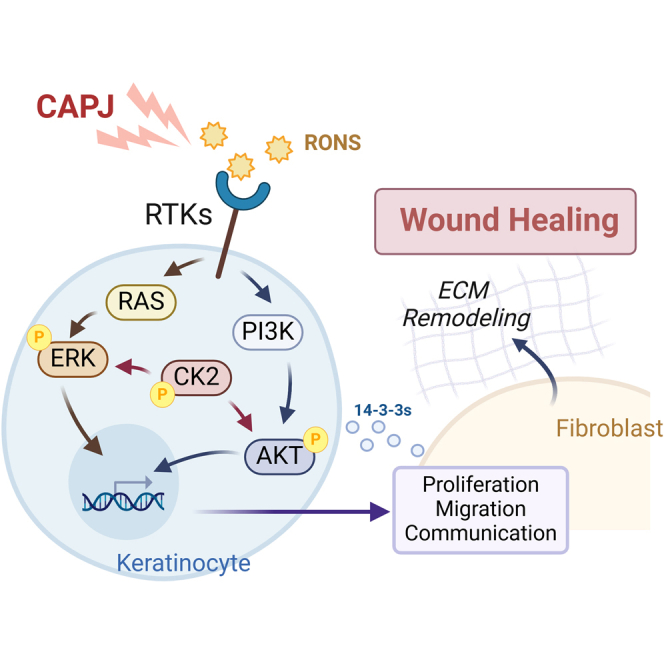

## In Brief

(Phospho)proteomics analyses have revealed that CAPJ enhances wound healing via CK2 coordinated PI3K/AKT and MAPK phosphorylation events, which boosts keratinocyte migration. Furthermore, CAPJ consistently influences the late phase of wound closure by upregulating 14-3-3 proteins expression, which facilitates keratinocyte-fibroblast cross talk and promotes ECM remodeling at wound area during epithelialization. This study unveils the molecular mechanisms behind CAPJ's therapeutic effects, offering valuable insights for better predictions and treatment outcomes.

Cold atmospheric plasma jet (CAPJ), also known as nonthermal or low-temperature plasma jet, is a device operated at atmospheric pressure and near room temperature (<40 °C). It utilizes inert or reactive gases such as helium, argon, oxygen, nitrogen, air, or mixtures, exposed to a strong voltage or electric field to generate discharge. This process produces various reactive species, including free radicals, electric fields, charged particles, UV photons, and reactive oxygen and nitrogen species (RONS) (1, 2). The clinical utility of CAPJ lies on its ability to deliver reactive particles into cells, tissues and liquids in a noncontact, painless, and harmless manner (3). Therefore, the therapeutic applications of CAPJ have rapidly developed in various medical specialties, including oncology (cancer therapy) (4), dentistry (biofilm inactivation and bactericidal effect) (5, 6, 7), neurology (enhancing cell differentiation) (8), dermatology (wound healing and blood coagulation) (9, 10, 11), highlighting the favorability of CAPJ to be an innovative, safe, and promising treatment strategy.

In the context of skin wound healing, CAPJ has demonstrated its capability to enhance cell proliferation and migration both *in vitro* and *in vivo* studies (12, 13). Moreover, clinical investigations have shown that CAPJ can promote wound repair and reduce microbial load in patients with acute or chronic ulcerative wounds (14, 15, 16). Wound healing is a complex and tightly regulated processes involving a synchronized cascade of events across various cell types, including keratinocyte, fibroblast, and immune cells, four overlapping yet distinct phases (hemostasis, inflammation, proliferation, and remodeling), and intricate interaction among signaling molecules (17, 18, 19). RONS, such as H_2_O_2_, OH, NO, and NO_2_^-^, play crucial roles as pleiotropic signaling agents during wound closure, coordinating immune cell recruitment, regulating angiogenesis and blood perfusion, facilitating cell division and migration, as well as minimizing microbial infection (20, 21, 22). As CAPJ provides an exogeneous and tunable source of RONS, it has the potential to modulate intracellular RONS concentration, thereby influencing signal transduction at various cellular regulation levels during the wound healing process (23, 24). However, the detailed molecular mechanisms driving CAPJ-mediated wound healing remain largely unexplored, necessitating further research to elucidate the underlying signaling modulations for practical biomedical applications.

In this study, we aimed to characterize the molecular mechanisms underlying wound healing modulated by CAPJ using parallel mass spectrometry (MS)-based proteome and phosphoproteome analyses with human keratinocyte, specifically HaCaT cells, as the cell model. The combined proteomic and phosphoproteomic approaches provided a comprehensive overview of relative protein abundance and phosphorylation dynamics, offering insights into vast kinase-substrate networks (25) and perturbation of signaling pathways (26). In brief, HaCaT cells were indirectly treated with CAPJ via exposing to plasma-activated medium (PAM), following established procedures (12). The integrated proteomic data were systematically analyzed to dissect the altered signaling pathways in keratinocytes upon PAM intervention. Our (phospho)proteomic results revealed that CAPJ intervention significantly activated extracellular signal-regulated kinase (ERK), casein kinase 2 (CK2), and serine/threonine-protein (AKT) kinases, leading to disturbances in phosphorylation-dependent signaling cascades such as PI3K/AKT and mitogen-activated protein kinase (MAPK) pathways. Notably, CK2 was identified as a key coordinator of these perturbed pathways. Further verification experiments confirmed that CAPJ can promote cell migration and extracellular matrix (ECM) protein production during the late phase of wound healing. Collectively, these insights provide a deeper understanding of how CAPJ accelerates wound closure and offer valuable implications for the development of clinical treatments for skin wounds.

## Experimental Procedures

### Reagents and Antibodies

Chemical reagents included sodium deoxycholate (SDC), sodium lauroyl sarcosine (SLS), Tris-base, triethylammonium bicarbonate (TEABC), tris(2-caboxyethyl)phosphine hydrochloride, 2-chloroacetamide, TFA, chloroform (CHCl_3_), methanol (MeOH), ethanol (EtOH), acetonitrile (ACN), acetic acid (AA), EDTA, iron (III) chloride (FeCl_3_), ammonium phosphate (NH_4_H_2_PO_4_), MS-grade formic acid (FA) and phosphatase inhibitor cocktail 2/3 were obtained from Sigma-Aldrich. Protease inhibitor cocktail (EDTA-free) was purchased from BioTool, urea was obtained from Merck Millipore, and paraformaldehyde solution 4% in PBS was purchased from Santa Cruz. The BCA (bicinchoninic acid) protein assay kit was obtained from Thermo Fisher Scientific. Modified sequencing-grade trypsin and MS-grade Lys-C (lysl endopeptidase) were purchased from Promega and Wako, respectively. SDB-XC (polystyrene-divinylbenzene copolymer) Empore disks were obtained from VWR (Randor). Nickel nitrilotriacetic acid (Ni-NTA) agarose beads were purchased from QIAGEN. Polyvinylidene difluoride (0.45 μm pore size) membranes and enhanced chemiluminescent substrate were gained from PerkinElmer. For cell culture, Dulbecco’s modified Eagle’s medium (DMEM), fetal bovine serum, Penicillin–Streptomycin 100X solution were all obtained from HyClone. The primary antibodies for immunoblotting analysis were shown as follows: p44/22 MAPK (Erk1/2, #4695S), pT202/Y204-p44/22 MAPK (Erk1/2, #4370S), phosphoinositide 3-kinase (PI3K) (p110α, #4249S), pS473-AKT (#4060S), AKT (#4691S), pS2448-mTOR (#2972S), mTOR (#2971S), 14-3-3η (#5521), and 14-3-3γ (#5522) were purchased from Cell Signaling. CK2 beta (A301–984A) and pS209-CK2 beta (441090G) were obtained from Bethyl Laboratories and Thermo Fisher Scientific, respectively. CTNNB1 was purchased from BD Biosciences (610153). β-actin was obtained from abcam (ab8227). GAPDH (AC001) and α-tubulin (AC007) were purchased from ABclonal). The secondary rabbit horseradish peroxidase-conjugated antibody (RA-BZ202) was obtained from Croyez Bioscience.

### Cell Culture

Human keratinocytes (HaCaT, catalog number: PCS-200–011) were cultured in DMEM containing 10% decomplement fetal bovine serum, 100 U/ml penicillin and 100 mg/ml streptomycin. The cells were maintained at 37 °C in a humidified air with 5% CO_2_.

### Preparation of PAM

An indirect treatment of CAPJ applied for HaCaT cells were operated according to previous report (12). The DMEM medium was activated by CAPJ before usage with the parameters as follows: the working voltage was set as 6.5 kV and the distance between CAPJ and DMEM surface was 15 mm; the gas mixture of He and Ar with the flow rate of 5 and 0.5 slm, respectively, was used, and the treatment period was 15s. The temperature of DMEM surface was maintained at 34.5 °C during CAPJ treatment. The PAM was fresh prepared before cell culture experiments.

### Protein Extraction and Digestion

For “receiving” proteome and phosphoproteome, HaCaT cells cultured in 6-cm dish were replaced with normal medium or PAM for 2 h treatment, these cells were collected for receiving (phospho)proteome analysis. For “recovery” proteome analysis, both control and PAM-treated cells are replaced with normal medium and further incubated for an additional 24 h. Treated cells were washed with ice-cold PBS and lysed on-dish with 200 μl/well of lysis buffer contained 12 mM SDC, 12 mM SLS, 100 mM Tris–HCl (pH 9.0), protease, and phosphatase inhibitors. Cell with lysis buffer was incubated on-ice for 1 to 2 min, and then cell lysate was immediately collected by using cell scraper. Cell lysates were first heated at 95 °C for 5 min, homogenized with Bioruptor Plus sonicator (Diagenode) at 4 °C for five cycles (30 s on/off, low-mode) and centrifuged at 13,500 rpm for 30 min under 4 °C. The supernatant was transferred into a clean tube and subjected for MeOH/CHCl_3_ protein precipitation. The protein pellet was collected by centrifugation using 13,500 rpm for 10 min at room temperature and dissolved in 8 M urea in 50 mM TEABC. The concentration of protein was measured by BCA protein assay. The protein extract was subsequently reduced with 10 mM tris(2-caboxyethyl)phosphine hydrochloride for 30 min and alkylated with 50 mM 2-chloroacetamide for 45 min at 29 °C. Samples were diluted with 50 mM TEABC prior to digestion with Lys-C (1:100, w/w) for 3 h followed by trypsin (1:50, w/w) for overnight at 29 °C. Digested peptide sample was acidified with TFA and desalted with homemade StageTip packing with SDC-XC membrane. The peptides were dried with vacuum centrifugation and stored in −80 °C until liquid chromatography tandem mass spectrometry (LC-MS/MS) analysis.

### Phosphopeptide Enrichment

The phosphopeptides were enriched by Fe^3+^-immobilized metal affinity chromatography as described in previous study (27). Briefly, the nickel nitrilotriacetic acid agarose beads were suspended with 6% AA (pH 3.0) and loaded into a microtip packaged with a frit disk capped in the end, then deactivated with 50 mM EDTA in 1M NaCl. Then the beads were equilibrated with 6% AA, activated with 100 mM FeCl_3_ in 6% AA and equilibrated with 6% AA again. Following, 200 μg of desalted peptides per sample were dissolved in 80% ACN contained 0.1% TFA, loaded into the preactivated microtip, and then washed twice with 80% ACN contained 0.1% TFA and 1% AA. The phosphopeptide was eluted with 200 mM NH_4_H_2_PO_4_ and desalted with homemade StageTip packed with C18 membrane. Phosphopeptide sample was dried by using vacuum centrifugation and dissolved in 0.1% FA before LC-MS/MS analysis.

### LC-MS/MS Analysis

The tryptic peptides and enriched phosphopeptides were analyzed with Orbitrap Fusion Lumos Tribrid MS coupled with UltiMate 3000 RSLCnano system (Thermo Fisher Scientific). The peptide mixture was loaded onto Thermo Scientific PepMap C18 column (25 cm × 75 μm ID) and separated at a flow rate of 300 nl/min through a 120 min gradient from 4% to 38.5% buffer B (ACN with 0.1% FA) for proteome and 6% to 50% buffer B for phosphoproteome. Buffer A was H_2_O contained 0.1% FA. The column temperature was set at 45 °C. The MS was operated in data-dependent acquisition with Top-Speed mode for a cycle time as 3 s. For proteome, survey full-scan MS spectra were acquired in orbitrap (*m/z* 375–1500) with a resolution of 60 K, automatic gain control (AGC) target at 4e5 and auto maximum injection time; for phosphoproteome, MS scans were acquired in orbitrap with *m/z* 400 to 1250 and the resolution was set at 60K, AGC target at 4e5 and maximum IT was 50 ms. The most intense ions were sequentially isolated for higher energy collision dissociation (HCD) MS/MS fragmentation and detected in the orbitrap with dynamic exclusion for 20 s. For both proteome and phosphoproteome, the MS/MS parameters were set as follows: a resolution of 30K, an isolation window of 1.4 Th with auto tuned of AGC target and injection time. Fragmentation was performed with normalized collision energy of 30%. Advanced peak detection function is on with a precursor fit threshold of 70% at 1.4 *m/z* window. The precursor ions with 2+ to 7+ charges were selected for HCD fragmentation.

### Data Processing

The raw MS/MS data were processed for peptide and protein identification and quantitation using MaxQuant (v1.6.14.0) by Andromeda search engine against Swiss-Prot *Homo sapiens* database (download on 2020.05.19) containing 26,561 protein entries. The search parameters were set as follows: full tryptic digestion allowing up to two missed cleavages; oxidation (M, +15.995 Da), acetylation (protein N-term, +42.011 Da), and phosphorylation (STY, +79.966 Da) were set as variable modifications, carbamidomethyl (C, +57.0214 Da) was set as a fixed modification. The mass tolerance was set as 10 ppm and 0.05 Da for precursor and fragment ions, respectively. The false discovery rate (FDR) filtration was set at 1% for both the peptide and protein levels. Proteome and phosphoproteome were both quantified using label-free strategy, and the abundance was further analyzed by Perseus (v 1.6.15.0).

### Bioinformatics Analysis

The 13-mer sequence window surrounds the phosphosite (±6) obtained from the MaxQuant search result was used to identify their kinases and substrates by using PHOXTRACK (PHOsphosite-X-TRacing Analysis of Causal Kinases) (28). The list of sequence tags and their corresponding log2 ratios of PAM and Ctrl were utilized as input. Kinase prediction was performed using PHOXTRACK with default settings, including 50,000 permutations, a minimum number of three substrates, and unweighted statistics. The pLogo generator (v1.2.0) (29) and PhosphoSitePlus (30) was used for sequence motif analysis. Kyoto Encyclopedia of Genes and Genomes (KEGG) pathway enrichment and Gene Ontology functional annotation were performed using DAVID Bioinformatics Resources (v2023q2) (31). The Reactome pathways were enriched on Reactome Pathway Database (v84) (32). All enrichment significance FDRs were calculated using Benjamini–Hochberg approach. For network analysis, the topology was constructed using STRING (v11.5) (33) and analyzed in Cytoscape (v3.8.2) (34).

### Immunoblotting Analysis

For inhibition experiments, HaCaT cells were treated with normal medium, PAM, SCH772984 (200 nM, ERK inhibitor) or CX4945 (10 μM, CK2 inhibitor), and cotreatment of PAM and inhibitor for 2 h. For CAPJ long-term influence evaluation, PAM-treated HaCaT cells were cultured for additional 24 and 48 h. Then, cells were lysed using 12 mM SDC, 12 mM SLS in 100 mM Tris–HCl (pH 9.0) contained protease and phosphatase inhibitors. Protein concentration was measured by BCA protein assay. Equal amounts of protein from each sample was analyzed by 10% SDS-PAGE followed by transfer to a polyvinylidene difluoride membranes. Membrane was blocked with 5% bovine serum albumin in Tris-buffered saline containing 0.1% Tween-20 (TBST), probed with indicated primary antibodies (1/1000–1/3000, v/v), detected with horseradish peroxidase-conjugated secondary antibodies (1/5000, v/v), visualized by enhanced chemiluminescent substrate, and imaged using ChemiDoc Imaging System (Bio-Rad Laboratories). The quantitative densitometry of immunoblot was performed by using ImageJ 1.53t (National Institutes of Health, USA).

### Cell Migration Assay

HaCaT cells were cultured in 12-well plates at a density of approximately 1 × 10^5^ cells/ml and incubated for 24 h to reach around 100% grown confluence. Then, a sterile p200 pipette tip was used to scratch the cell monolayer across the center of each well. The detached cells were removed by washing with PBS. The remaining adherent cells were treated with normal medium, PAM, SCH772984 (200 nM, ERK inhibitor) or CX4945 (10 μM, CK2 inhibitor), and cotreatment of PAM and inhibitor for 2 h. Following, the medium was replaced to normal medium again and incubated for additional 24 h. The microscopic images of wounded area were monitored by optical microscopy. The image of wounded area was measured by using ImageJ. The wound closure ratio (%) was calculated using $\frac{Wi-Wf}{Wi}\;x\;100$, where *Wi* and *Wf* are initial and final wound area, respectively.

### Determination of Nitric Oxide Concentration

The transient and volatile nature of nitric oxide (NO) render its detection challenging. Instead, total concentration of its stable metabolites, nitrite (NO_2_^-^) and nitrate (NO_3_^-^), are used to indicate the levels of nitric oxide (NOx) *in vivo*. NOx concentrations in ddH_2_O with and without PAM treatment for 15 s were detected using a commercial kit (Enzo Life Sciences). The absorbance was measured at 540 nm using a plate reader (SpectraMax iD3).

### Histological Analysis and ELISA

The cutaneous wounded male Sprague-Dawley rats obtained from the National Laboratory Animal Center of Taiwan were used to evaluate CAPJ treatment. The rates were treated in accordance with the Animal Care and Use Guidelines for Chang Gung University under a protocol approved by the Institutional Animal Care Use Committee (Approval No. CGU105-032).

Two full-thickness wounds with 17 mm in diameter were produced on both sides of a rat’s shoulders, in which right-side wound was treated with CAPJ and the left side wound was kept untreated as controls for comparison. He/Ar-CAPJ treatment was operated according to previous report (12), the working voltage, sample distance, and treatment period were 7.5 kV, 20 mm, and 60 s, respectively. Rats were euthanized on day-3, -7, and -14 after CAPJ treatment. For histological analysis, the cutaneous wounded tissues were harvested and fixed with paraformaldehyde solution 4% in PBS, dehydrated in ethanol, and embedded in paraffin. Tissue section (5 μm thick) was prepared in a standard fashion and observed in detail using Masson’s Trichrome Stain kit (StatLab, #KTMTR), Elastic Stain Kit (Modified Verhoeff's, ScyTek, ETS-1-IFU) and Alcian Blue (pH 2.5) Stain Kit (ScyTek, AFR-1-IFU). The stained section was photographed by using ZEISS Axio Imager M2 Microscope (Zeiss). Interleukin-1β and tumor necrosis factor-alpha levels for wound site tissues were determined by ELISA, following the manufacturer’s instruction (Cusabio). The absorbance was detected at 450 nm using SpectraMax iD3 plate reader. Results were calculated using second-order polynomial regression analysis.

### Experimental Design and Statistical Rationale

The proteome and phosphoproteome datasets were performed with three biological replicates for each experimental groups and duplicate LC-MS/MS runs were conducted for each sample. To identify differentially expressed proteins (DEPs) and phosphorylated residues, the label-free abundances were log2-transformed and further analyzed by Perseus for data filtering (100% valid value in at least one group), imputation (replacing missing values from normal distribution), and statistical evaluation such as principal component analyses, hierarchical analysis, and two sample *t*-tests (35). To validate our findings from (phospho)proteomic results, immunoblotting, cell migration assays, and histological analysis were performed with at least three independent experiments and presented as the bar plot with mean ± SD. Differences between experimental groups for both proteome and phosphoproteome analyses, as well as biological validations were performed by using two sample *t* test to determine the statistical significance, and the threshold was set as *p*-value <0.05 without applying statistical corrections.

## Results

### CAPJ Notably Disturbs Protein Phosphorylation Homeostasis Instead of Protein Turnover

**Fig. 1:**
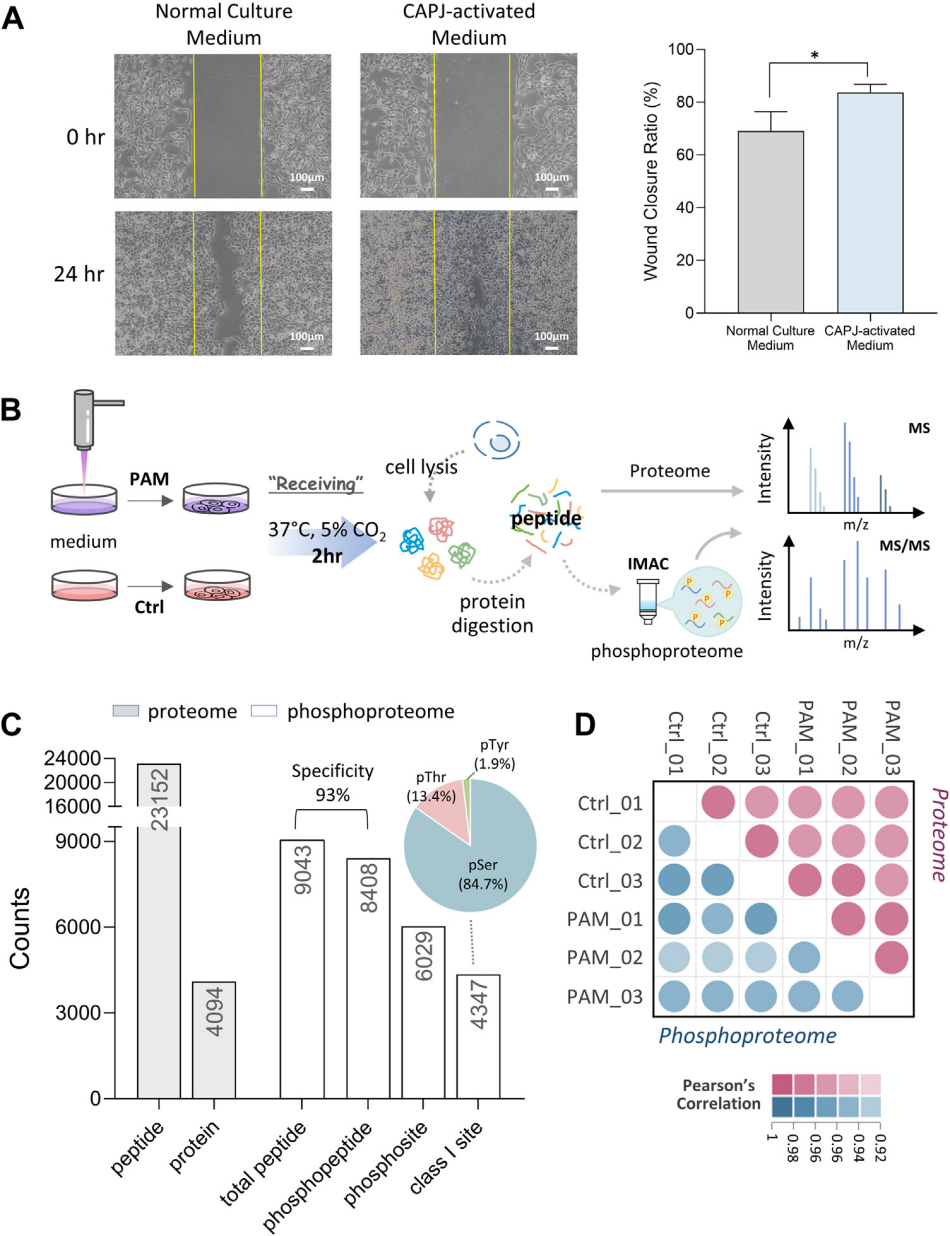
**Characterization of proteome and phosphoproteome in 2-h PAM-treated HaCaT cells.***A*, wound healing assay of HaCaT cells treated for 2 h with or without PAM, following a 24 h incubation. The wound closure ratio (%) is calculated using ImageJ and showed as means ± SD (*N* = 3). The significance is calculated using two sample *t* test (∗*p*-value <0.05). *B*, workflow of label-free quantitative (phospho)proteomics analysis. *C*, bar chart shows the identification number of peptide, protein, phosphopeptide, and class I phosphosite from biological triplicate. *Gray bars* indicate the number from proteome analysis, while *white bars* are from phosphoproteome analysis. Pie chart showed the distribution of class I phosphosites on serine (Ser), threonine (Thr), and tyrosine (Tyr) residues. *D*, dot plot presents the quantitative reproducibility of proteins (*red*) and phosphosites (*blue*) between biological triplicates. The color indicated the value of Pearson’s correlation coefficient. PAM, plasma-activated medium.

We first established our CAPJ-treated cell model by exposing HaCaT cells to PAM for 2 h; indeed, the wound healing assay showed that the ability of cell migration was clearly enhanced in the CAPJ-intervened group compared to the control (Fig. 1*A*). To dissect how PAM induces cell migration, label-free quantitative proteome and phosphoproteome analyses were performed in parallel, as illustrated in Figure 1*B*. In proteome analysis, a total of 23,152 unique peptides from 4094 protein groups were identified with 1% FDR at both peptide and protein levels (Fig. 1*C* and Supplemental Table S1). Following phosphopeptide purification, the enrichment specificity reached up to approximately 93%, and we identified a total of 6029 phosphosites from 8408 phosphopeptides (Fig. 1*C*). A distribution of phosphoserine (pSer), phosphothreonine (pThr), and phosphotyrosine (pTyr) at 84.7%, 13.4%, and 1.9%, respectively (Fig. 1*C*) were revealed among 4347 unambiguously localized phosphosites. High Pearson’s correlation coefficient within biological triplicates, ranging from 0.97 to 0.99 for proteome (red dots) and 0.91 to 0.95 for phosphoproteome (blue dots), indicated the good reproducibility across these datasets (Fig. 1*D*). Furthermore, the coefficient of variation (%) showed that more than 95% of proteins and 85% of phosphosites had the coefficient of variation less than 5% (Supplemental Fig. S1*A*), once again demonstrating the good quantitative reproducibility and accuracy of our (phospho)proteomics analysis. The log2 transformed fold-changes (FCs) between PAM and Ctrl for both the proteome and phosphoproteome showed a normal distribution with up to 19% of the quantified phosphosites displayed a FC ≥ 2, while only 8% (266 proteins) among the quantified proteins (Supplemental Fig. S1*B*, Supplemental Tables S1 and S2). Furthermore, significant changes (*p*-value <0.05 without correction) were observed, affecting 173 proteins (Supplemental Fig. S1*C*, Supplemental Table S1) and 435 phosphorylated residues from 341 phosphoproteins (Supplemental Fig. S1*D*, Supplemental Table S2). These results suggest that keratinocyte migration induced by PAM was initially driven by phosphorylation-mediated signaling, rather than by changes in protein turnover. Interestingly, only 10 differential features were commonly found between the proteome and phosphoproteome (Supplemental Fig. S1*E*), underscoring the distinct effects of PAM on protein expression and phosphorylation. This highlights the need for a multilayered analysis to fully understand the molecular mechanisms at play. Collectively, the robustness of our (phospho)proteomic data provided a foundation and has sparked our interest in exploring the mechanisms behind PAM-induced cell migration, with a particular focus on protein phosphorylation changes in early cellular responses to PAM intervention.

### CAPJ Activates PI3K/AKT and MAPK Signaling Cascades to Promote Wound Healing

A total of 4366 quantified class I phosphosites were included in principal component analysis (PCA), which revealed a distinct separation between the Ctrl and PAM groups along the first principal component, with PC1 and PC2 explaining 30.5% and 20.4% of the total variance, respectively. Furthermore, all triplicate fell within the 95% confidence interval boundaries for each group, demonstrating good reproducibility across biological replicates (Supplemental Fig. S2*E*). Additionally, the hierarchical clustering demonstrated effective grouping of biological triplicates, underscoring the good reproducibility of this dataset. The 435 regulated phosphosites were clustered into two groups with 150 phosphosites being upregulated and 285 being downregulated in response to PAM stimulation (Supplemental Fig. S2*B*: heatmap and Supplemental Table S2). These 435 altered phosphosites were from 350 proteins and significantly participated in the pathways of PIP3 activation of AKT signaling, PTEN regulation, and cell junction organization (Supplemental Fig. S2*B*: bar char and Supplemental Table S6; Benjamini-Hochberg, FDR <0.05), which are pivotal in the downstream regulation of cell growth and movement (36, 37). Furthermore, the intracellular distribution of these regulated phosphoproteins, primarily spanning from the cytosol to the nucleus and the annotated biological processes involved in DNA replication and transcription (Supplemental Fig. S2*C*, Benjamini-Hochberg, FDR <0.05), align with the concept that transcriptional regulators rely on kinases/phosphatases-mediated protein phosphorylation for nuclear translocation and orchestrating signaling cascades (38, 39). Though PAM treatment simultaneously induced alterations at both protein expression and phosphorylation levels (Supplemental Fig. S1, *B*–*D*), thereby enhancing keratinocyte migration and growth, it is evident that protein phosphorylation is more significantly affected in the initial intracellular regulation.

**Fig. 2:**
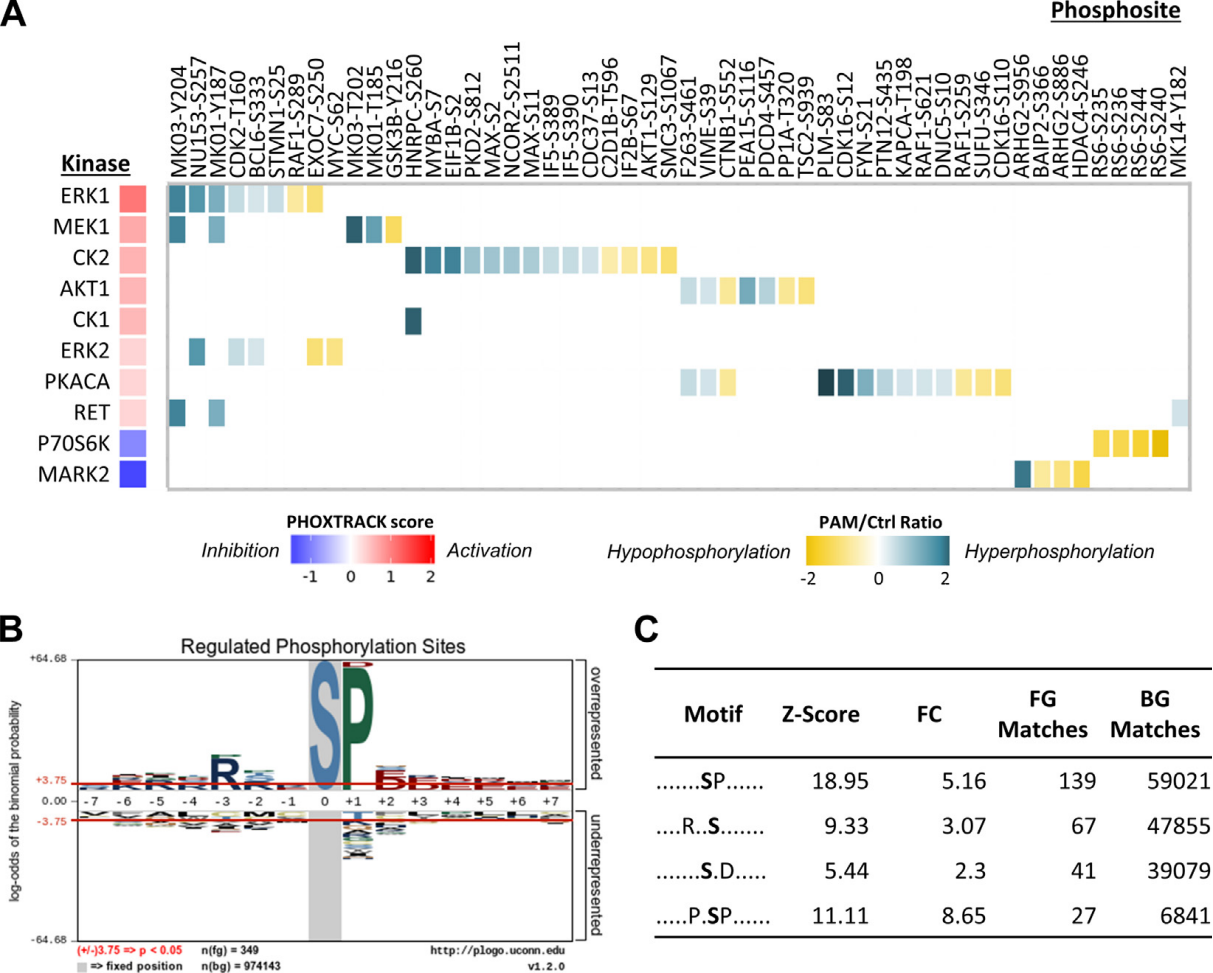
**Phosphorylation sites reveal preferential signaling pathways activated by 2 h PAM treatment in HaCaT cells.***A*, heatmap presents the predicted kinases and their specific substrates in phosphoproteome analysis. The color code of PHOSTRACK score (*red*-*blue*) reflected the degree of activation or inhibition of kinase activity, while the color code of PAM/Ctrl ratio displayed the change degree of phosphorylated substrates for each kinase and their distribution over the hypophosphorylated (*yellow*) and hyperphosphorylated (*azure*). *B*, motif analysis of significantly regulated phosphoserine residues (349 sequence windows) using pLogo. *X*-axis is the positions of the amino acid residues at C terminal or N terminal to the central phosphoresidue (position “0”). *Y*-axis is the proportional height of the amino acid residues enriched at the specific position in the pool of the queried phosphopeptides. The *red horizontal bars* on the pLogo correspond to *p* = 0.05. *C*, table represents the scoring information of Top 4 enriched motifs analyzed by using PhosphoSitePlus. Z-score indicates the significance of converted *p*-value (<1e-06). Fold-change (FC) reflects the enrichment of sequence window entities as compared to the background population of motifs. “Foreground matches” refers to the number of matches within the input entities (FG matches), while “background matches” denote the number of matches within the background (BG matches). FC is calculated by dividing (FG matches/FG size) by (BG matches/BG size), where FG size is the number of our input sequences for pSer (366), and BG size is the total number of motifs in database (802,214). PAM, plasma-activated medium.

Protein kinases serve as central components in mediating protein phosphorylation, responsible for the regulation of various cellular processes in response to environmental stimuli. To figure out the crucial factors of PAM intervention, the surrounding sequences (±6) of class I phosphosites, along with the information of log2 ratio between PAM and Ctrl, were submitted to PHOXTRACK for predicting the kinase activity (28). Clearly, 10 protein kinases were significantly enriched, with eight kinases activated and two inhibited upon PAM treatment (Fig. 2*A*). Notably, a majority of hyperphosphorylated events that showed significant phosphorylation differences after PAM treatment was identified as substrates of CK2 and ERK1/2. Among the eight activated kinases, ERK1/2 and MEK1 are integral components of the MAPK signaling pathway. Together with AKT1, they are recognized as diverse downstream regulators of receptor tyrosine kinase (RTK) signaling, modulating a myriad of biological processes to mediate cell proliferation and migration (40, 41). Furthermore, the two main casein kinases, CK1 and CK2, were highly activated, playing evident roles in regulating diverse signaling pathways for normal cellular function, including intracellular trafficking, apoptosis, cell growth, and metabolism (42, 43, 44). In addition, CK2 acts as a pivotal modulator, intervening in both the PI3K/AKT and MAPK pathways. It directly phosphorylates AKT at Ser129 to enhance its activity and simultaneously inhibits the phosphatase activity of PTEN, ensuring the activation of PI3K/AKT signaling (44). Alternatively, CK2 can phosphorylate ERK activation loop, prompting ERK nuclear translocation for transcriptional regulation (45). PKACA, a subunit of protein kinase A (PKA), is known to intensify PI3K/AKT and MAPK pathways via modulating protein phosphatase activity (46), which was also found to be activated by PAM treatment (Fig. 2*A*). Additionally, activated RET can recruit and bind several proteins involved in PI3K/AKT, MAPK, JAK2/STAT3, and PLCγ pathways for signal propagation (47). The majority of substrates of CK2, ERK1/2, and MEK1 were hyperphosphorylated, suggesting PAM-induced activation of the PI3K/AKT and MAPK pathways in HaCaT cells (Supplemental Fig. S1*D*). Conversely, PAM treatment slightly suppressed the activity of P70S6K and MARK2 (Fig. 2*A*). P70S6K, a downstream kinase of the mTOR signaling pathway, regulates cell growth (48), while MARK2 is associated with focal adhesion by promoting FAK activation (49). However, their substrates only exhibited a slight reduction, and most of the hypophosphorylated events did not enrich specific kinases (Supplemental Fig. S1*D*). Consistent with these findings, pLOGO motif analysis revealed significant enrichment of phosphoserine motifs associated with proline-directed (proline at position +1), basophilic (arginine at positions −3 and −5), and acidic (glutamate/aspartate at positions +1 to +7) residues, which align with the motifs of ERK1/2, AKT1, and CK2 (Fig. 2*B* and Supplemental Fig. S3). Additionally, the top four motifs enriched by PhosphoSitePlus (30) corresponded to the recognition motifs for ERK (P-X-S/T-P) (50), AKT1 (R-X-R-X-X-T/S) (51), and CK2 (S-X-X-D/E) (44), with Z-scores ranging from 5 to 19 and FC values between 2 and 9 (Fig. 2*C*). These results confirm that PAM stimulation activates the PI3K/AKT and MAPK pathways via a series of phosphorylation events mediated by ERK, CK2, and AKT kinases.

### Kinase-Oriented Phosphoproteome Network Highlights the Involvement of PI3K/AKT and MAPK Pathways upon CAPJ Intervention

**Fig. 3:**
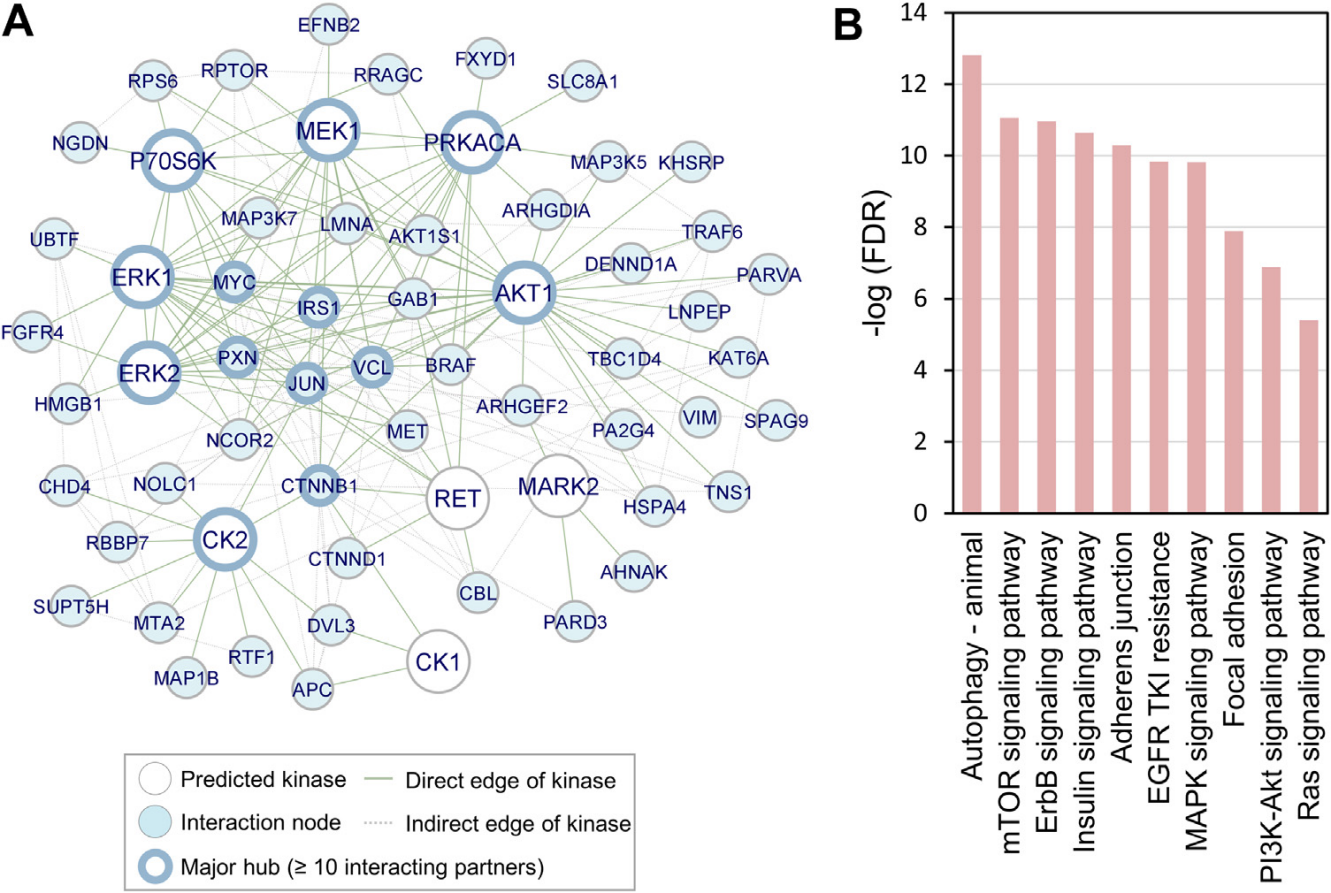
**Protein network of enriched kinases and proteins with regulated phosphosites in 2 h PAM-treated HaCaT cells.***A*, all the proteins with significantly regulated phosphorylation events (cyanite nodes, two sample *t* test, *p*-value <0.05 without correction) and predicted kinases (*white* nodes) are submitted to STRING for protein-protein interaction analysis. The interactors with direct interaction and predicted kinases are depicted on the network topology. *B*, the predicted kinases and their direct interactors in network topology are subjected for KEGG pathway mapping. Bar chart presents the -log10 transformed enrichment significance FDR, which are *p*-value corrected using Benjamini–Hochberg approach. FDR, false discovery rate; KEGG, Kyoto Encyclopedia of Genes and Genomes; PAM, plasma-activated medium.

The fact that protein phosphorylation often primes the formation of protein-protein interactions (PPIs), which are the backbone of signal transduction and lead to changes in protein conformation, localization, activity, and stability (26, 52), prompted us to explore the interplay between PAM-altered phosphorylation events through network topology. By incorporating 350 phosphoproteins with both upregulated and downregulated phosphosites, along with the 10 perturbed kinases, into the STRING database (33), 156 nodes and 974 edges, with 51 protein nodes showing direct interactions (124 edges) with the 10 predicted kinases were observed in the resulting network topology. Notably, most of these kinases interacted with one another, indicating promising regulatory signaling pathways and kinase-substrate interactions involved during PAM treatment (Fig. 3*A*). In general, nodes with 10 or more interacting partners are generally regarded as significant hubs, playing a vital role in regulating cellular processes (52). Among the kinases CK2, ERK1/2, AKT1, MEK1, and P70S6K each had over 10 interacting partners, serving as major hubs that align with the motif enrichment results (Fig. 2, *B* and *C*). Additionally, several phosphoproteins emerged as significant hubs, including PXN (paxillin), VCL (vinculin), CTNNB1 (catenin β1), JUN (transcription factor Jun), IRS1 (insulin receptor substrate 1), and MYC (Myc proto-oncogene protein). PXN, VCL, CTNNB1, and JUN are focal adhesion proteins that participate in regulating cell-ECM adhesion (53) and keratinocyte movement during wound healing (54). Furthermore, IRS1 is crucial for the nuclear translocation of CTNNB1 and the activation of MYC transcription, which induces the PI3K/AKT and MAPK signaling pathways (55). These significant hubs demonstrated diverse interactions with the majority of predicted kinases, suggesting their phosphorylation is subject to multiple regulatory mechanisms.

Moreover, most kinases interacted directly with each other and were involved in PI3K/AKT and MAPK signaling pathways. In contrast, CK2 exhibited indirect interactions with other kinases via CTNNB1 or JUN, suggesting that CK2 acted as a coordinator within PI3K/AKT and MAPK signaling pathways, thereby indirectly influencing cell migration outcomes (Fig. 3*A*). Although previous studies have shown that CK2 can directly phosphorylates ERK and AKT (43, 44), this was not evident in our PPI analysis, underscoring the need for a comprehensive database to facilitate the integration of protein phosphorylation and interactions. Furthermore, Kyoto Encyclopedia of Genes and Genomespathway analysis revealed that the 61 interacting modules were significantly enriched in pathways related to autophagy, ErbB signaling, PI3K/AKT/mTOR signaling, MAPK signaling, and cell adhesion (Fig. 3*B* and Supplemental Table S7). This suggests their involvement in the RTK network pathway, which plays a crucial role in modulating cell growth and movement (56, 57). Collectively, phosphoproteomics analysis together with PPI network affirmed the significant activation of PI3K/AKT and MAPK signaling in PAM-treated HaCaT cells. Keratinocytes are well-known to play a key role in epithelialization during the wound closure process through activating RTK signaling pathways for cell differentiation, proliferation, migration, and cell-cell communication to achieve epidermal restoration (58, 59). This corresponds with our observation that CAPJ stimulated PI3K/AKT and MAPK signaling, thereby accelerating keratinocyte migration during wound healing. In sum, the integrated analysis of protein phosphorylation and PPIs highlights the significant role of CAPJ in mediating the molecules and mechanisms that drive wound healing in keratinocytes.

### CK2 Coordinates PI3K/AKT and MAPK Pathways in CAPJ-Facilitated Wound Healing

**Fig. 4:**
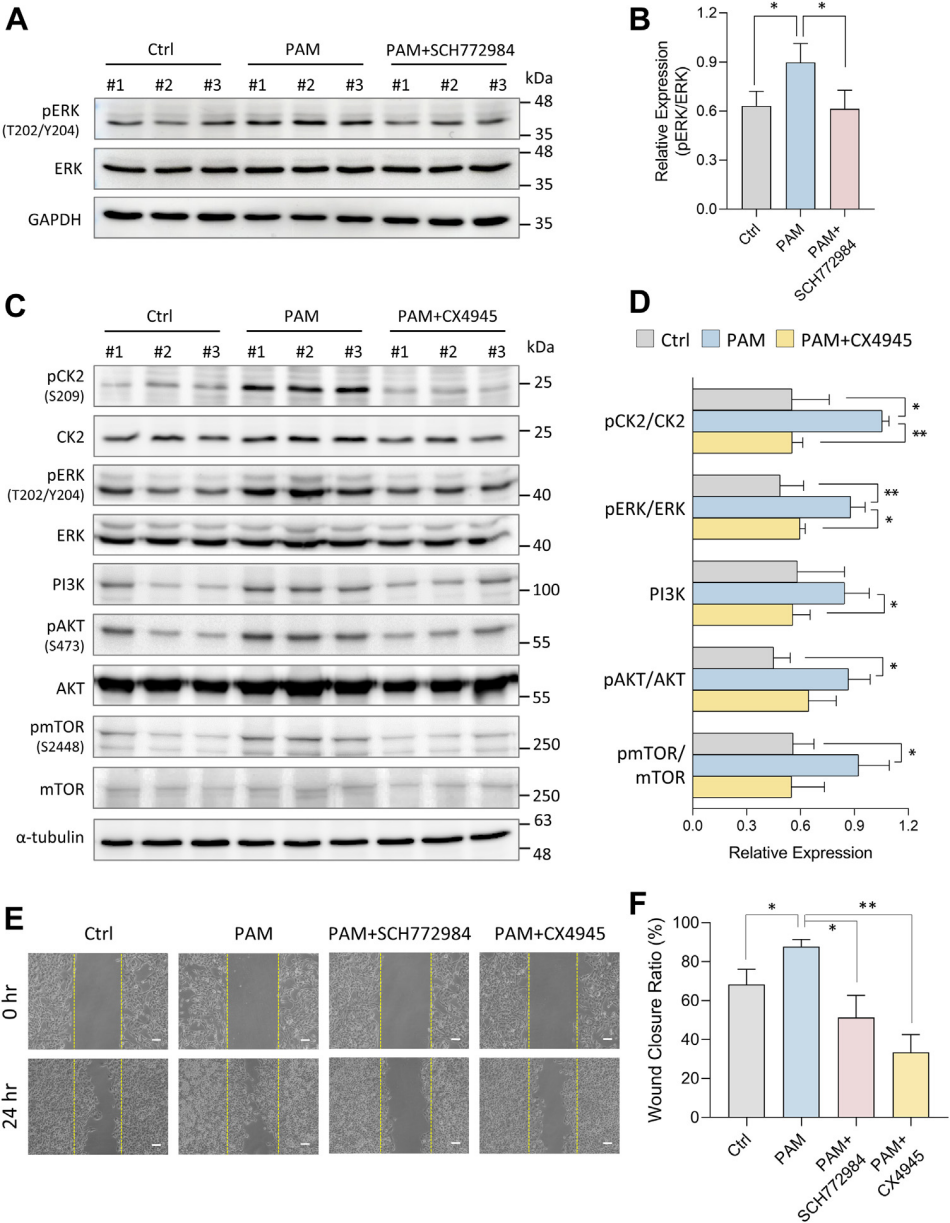
**PAM-induced activation of PI3K/AKT and MAPK signaling in HaCaT cells.** Cells are treated with PAM alone or cotreated with PAM and (*A* and *B*) ERK inhibitor (SCH772984), or (*C* and *D*) CK2 inhibitor (CX4945) for 2 h. Cell lysates are collected for immunoblotting assay against indicated antibodies, accompanying with a control group (Ctrl) treated with normal medium. Bar charts of (*B*) and (*D*) represent the densitometry analyses using ImageJ for immunoblots of (*A*) and (*C*), respectively, and expressed as phosphoprotein/total protein ratio. Expression level of PI3K is normalized with α-tubulin. *E*, representative images of wound healing assay in sample groups of Ctrl, PAM, PAM + SCH772984, and PAM + CX4945 for 0 h and 24 h incubation. *Yellow dotted lines* represent the wound boundary. The scale bar represents 100 μm. *F*, bar chart illustrated the wound closure ratio (%) of (*E*). Data were showed as means ± SD of independent triplicate. All significance was calculated using two sample *t* test. ∗*p*-value <0.05, ∗∗*p*-value <0.01. AKT, serine/threonine-protein kinase; CK2, casein kinase 2; MAPK, mitogen-activated protein kinase; PAM, plasma-activated medium; PI3K, phosphoinositide 3-kinase.

Based on our findings and literature reviewing, we hypothesized that ERK, AKT, and CK2 kinases are the key responders to PAM treatment, subsequently activating the PI3K/AKT and MAPK signaling pathways to promote cell migration (50, 60, 61) (Figs. 2 and 3). To validate this hypothesis, we administered SCH772984 (62), a selective ERK1/2 inhibitor, and CX4945 (63), a CK2 inhibitor, to PAM-treated HaCaT cells. As expected, SCH772984 treatment alone significantly reduced the phosphorylation levels of ERK at its activation sites (Thr 202/Tyr 204) to below control levels (Supplemental Fig. S5*A*), while PAM treatment led to an approximately two-fold increase in ERK phosphorylation (Fig. 4, *A* and *B*). Cotreatment with PAM and SCH772984 markedly suppressed both the enhanced phosphorylation and cell migration induced by PAM, reducing phosphorylation to control levels and cell migration to levels observed with SCH772984 alone (Fig. 4, *A*, *B*, *E* and *F*, and Supplemental Fig. S5*B*). Similarly, CX4945 effectively inhibited CK2 phosphorylation at Ser 209 and reduced cell migration as expected (Supplemental Fig. S5). In combined treatment of PAM and CK2 inhibitor, the CX4945 inhibitor significantly decreased CK2 phosphorylation to control level and reduced cell migration. Specifically, the wound closure ratio for CX4945 treatment alone was approximately 21%, while the combined treatment with PAM achieved a wound closure ratio of around 33%, representing a ∼1.5-fold increase compared to the inhibitor treatment alone (Fig. 4, *C*–*F*, and Supplemental Fig. S5). These findings suggest that, in addition to CK2, other pathways upstream of ERK activated by PAM may also contribute to enhancing cell migration, while CK2 plays a major role in reducing cell migration ability by more than 50%. Notably, CK2 inhibition also led to a reduction in PAM-induced ERK and AKT phosphorylation, along with decreased mTOR phosphorylation at Ser2448 and reduced expression of the PI3K catalytic subunit, which is critical for PI3K activity (64) (Fig. 4, *C* and *D*). These results are consistent with CK2’s regulatory role in modulating ERK and AKT activity via phosphorylation (44, 45), suggesting that CK2 coordinates the PI3K/AKT and MAPK signaling pathways in a bidirectional manner. Collectively, our finding insinuated that CAPJ promoted keratinocyte migration during wound healing through bidirectional activation of phosphorylation-dependent PI3K/AKT and MAPK signaling, with CK2 kinase acting as a key coordinator of this process.

### CAPJ Exhibits a Long-Term Influence on the Wound Healing Process

**Fig. 5:**
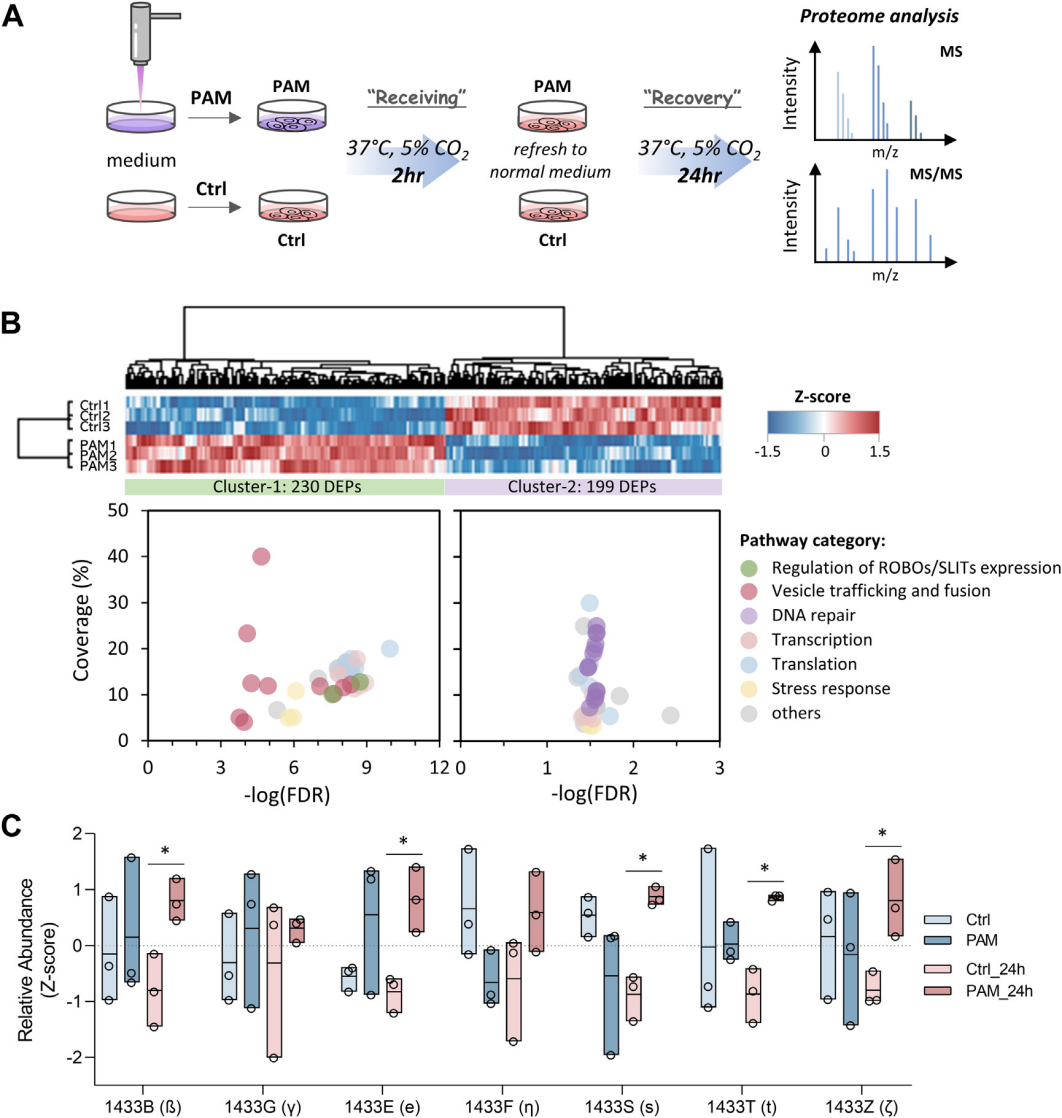
**Characterization of late-phase effects in PAM-treated HaCaT cells (recovery proteome).***A*, workflow of receiving and recovery proteome analyses. Cells are treated with PAM for 2 h (receiving), replaced with normal medium and incubated for additional 24 h (recovery). Protein extracts are subjected to digestion and the peptide sample was analyzed by LC-MS/MS. *B*, hierarchical clustering (*upper* panel) revealed two distinct clusters of differentially expressed proteins (DEPs, two sample *t* test, *p*-value <0.05). Proteins are clustered on the *top*, while samples are indicated on *left*. Color bar shows the Z-score normalized protein abundance. Pathway enrichment is performed using Reactome database and visualized with bubble plot (*lower* panel). *X*-axis is the pathway enrichment significance (FDR calculated using Benjamini–Hochberg approach) and *Y*-axis is pathway coverage (%). *C*, representative box plot with all points shows the relative abundance of 14-3-3 proteins compared between Ctrl (*light* color) and PAM (*dark color*) in receiving (*blue box*) and recovery (*red box*) proteomes. Two sample *t* test without correction was performed for differential significance. *Asterisk* symbol indicated the significant level (∗*p*-value <0.05). *Y*-axis was the protein abundance normalized with Z-score normalization. FDR, false discovery rate; LC-MS/MS, liquid chromatography tandem mass spectrometry; PAM, plasma-activated medium.

To examine the long-term influences of CAPJ intervention, two proteome analyses, the “receiving” and “recovery” proteomes, were conducted on keratinocytes. HaCaT cells were treated with PAM or control (Ctrl) for 2 h to assess the "receiving" proteome, followed by replacement with normal medium for an additional 24-h incubation as the "recovery" proteome in both groups (Figs. 1*B* and 5*A*). In total, 3223 quantified proteins were identified in the receiving proteome. PCA revealed good biological reproducibility within each group, with all samples falling within the 95% confidence intervals. The PCA plot showed that PC1 accounted for 47.4% of the variance, while PC2 explained 19.9%. Although the Ctrl and PAM groups were separated into two distinct clusters along PC2, this separation suggests that the differences between these two groups are less pronounced in the receiving proteome compared to phosphoproteome (Supplemental Fig. S4*A*), aligning with the result shown in Supplemental Fig. S1*B*. Next, hierarchical clustering showed that the 173 DEPs could be categorized into two distinct clusters (Supplemental Fig. S4*B*). Gene Ontology analysis of these DEPs revealed significant involvement in RNA metabolism, including RNA capping, transport, and modifications across various organelles, suggesting that PAM treatment may activate local mRNA translation and regulate protein synthesis (Supplemental Fig. S4*C* and Supplemental Table S8) (64). Although this receiving proteome dataset had showed the activation of early signaling and the initiation of protein synthesis in HaCaT cells treated with PAM for 2 h (Fig. 1*B*), the late-phase regulation of wound healing, which typically occurs days or weeks after injury (65), could not be observed from such dataset. To address this, we performed proteome analysis on HaCaT cells treated with PAM for 2 h and then incubated with normal medium for an additional 24 h, termed as the “recovery” proteome (Fig. 5*A*).

For the “recovery” proteome, out of 2824 quantified proteins, 230 DEPs were significantly upregulated (Cluster-1) with 199 DEPs downregulated (Cluster-2) after PAM treatment (two-sample *t* test, *p*-value <0.05 without correction, Fig. 5*B* heatmap and Supplemental Table S3). In contrast to the transcription, translation, and stress response, which were commonly enriched in both clusters, the pathways related to DNA repair were particularly enriched in downregulated proteins (Fig. 5*B*: right-side bubble plot for Cluster-2 and Supplemental Table S4), potentially associated with nuclear elongation caused by cell migration (66). Alternatively, pathways related to ROBOs/SLITs expression and vesicle trafficking and fusion were distinctly mapped among upregulated proteins (Fig. 5*B*: left-side bubble plot for Cluster-1 and Supplemental Table S5). ROBOs/SLITs signaling regulates cell proliferation and migration by controlling the intracellular localization of β-catenin (67) and has been implicated in promoting epithelial wound repair by extruding dead cells and inducing growth factors to injured tissues (68). Membrane vesicles, on the other hand, enhance wound healing by transferring various bioactive molecules between different cell types and mediating various signaling pathways in each phase of wound repairing (69). Of noted, 14-3-3 proteins were found to be involved in most pathways categorized into vesicle trafficking and fusion. Briefly, 14-3-3s are a family of structurally similar phospho-binding proteins consisting of seven isoforms (β, γ, ε, η, σ, τ, and ζ), known to regulate various cellular functions such as signaling, cell growth, and apoptosis (70). They are primarily involved in PI3K/AKT and MAPK pathways to modulate enzyme activity, control subcellular localization of interactors, and stimulate PPIs (71). Wound healing involves a complex interplay among various cell types, including immune cells, fibroblasts, keratinocytes, and endothelial cells, which primarily communicate through releasable factors, such as cytokines or growth factors (72). During wound epithelialization, keratinocytes can release stratifin (14-3-3σ) and its isoforms through exosomes, which stimulate dermal fibroblasts to migrate to the wound site, express matrix metalloproteinases, and promote the synthesis and reorganization of ECM proteins, particularly collagens (59). Comparing the receiving and recovery proteomes, we found that most 14-3-3 proteins were merely increased in the recovery set after PAM treatment (Fig. 5*C*), implying that the effect of PAM may persist into epithelialization phase during wound healing, facilitating wound closure via cross talk between keratinocytes and dermal fibroblasts.

### Persistent CAPJ Effects Improve ECM Remodeling During Epithelialization

Since wound closure typically takes several days (72) and PAM’s effects were found persisting to epithelialization phase, we sought to investigate the prolonged impact of CAPJ on keratinocytes. Clearly, PAM treatment sustained the activation of AKT, ERK, and CK2 kinases for up to 48 h, with a gradual decline over time (Supplemental Fig. S6, *A* and *B*). These suggested that PAM continuously boosts the PI3K/AKT and MAPK signaling pathways, thereby influencing the downstream cellular processes critical for wound healing, such as cell migration and communication. Furthermore, the observed increase in 14-3-3 proteins in the recovery proteome, along with the identification of CTNNB1 as a major hub for cell adhesion (Fig. 3*A*), prompted us to verify the prolonged effect of PAM. Indeed, the expression of both CTNNB1 and the 14-3-3 proteins (14-3-3η and 14-3-3γ) was elevated after 24 and 48 h culture (Supplemental Fig. S6, *A* and *B*). These results alluded that CAPJ exerts long-term influences on wound healing by consistently enhancing signal transduction, promoting cell movement, and even stimulating communication between different cell types.

**Fig. 6:**
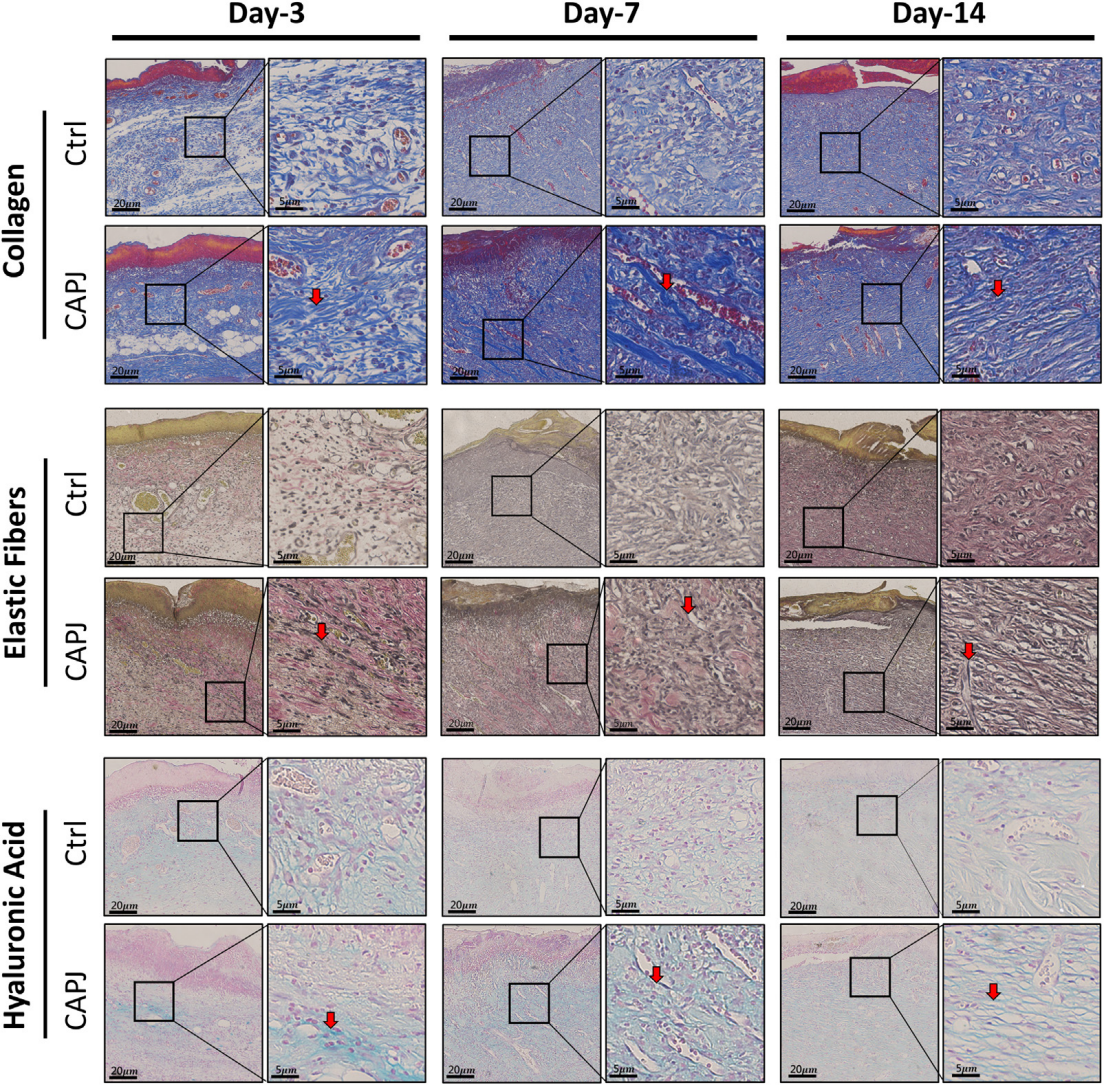
**Histological staining of extracellular matrix proteins in CAPJ-treated wound tissues.** Rat skin cutaneous wound was treated with CAPJ for 60s. CAPJ-treated wound tissue section (CAPJ), with parallel untreated control (Ctrl), were prepared on day-3, -7, and -14, and subjected for histochemical staining by using Masson’s Trichrome Stain kit for collagen staining (*top* panel, in *dark blue* color), Elastic Stain kit for elastin staining (*middle* panel, in *black* color), and Alcian *Blue* Stain kit for hyaluronic acid (HA) staining (*button* panel, in *light-blue* color). The *red arrows* were the extensively stained area for collagen and elastic fibers formation as well as the conjugates of HA and proteoglycans. *Right* panel correspond to high-magnification images of each cropped area on low-magnification image. Scale bars in low- and high-magnification images are 20 μm and 5 μm, respectively. CAPJ, cold atmospheric plasma jet.

The cross talk between keratinocyte and fibroblast during epithelialization is crucial for effective wound healing. Keratinocytes can influence fibroblasts through 14-3-3 proteins and other factors, encouraging the production, deposition, and reorganization of collagen and other ECM components within the wound microenvironment. ECM remodeling is another key determinant of successful wound healing, affecting both the speed of epithelialization and the quality of the newly formed tissue (73). To evaluate the CAPJ effects on ECM remodeling during wound repair, histological analysis of collagen, elastin, and hyaluronic acid, which are main ECM component of normal skin, were performed on rat skin tissue wounds at days 3, 7, and 14 post-CAPJ treatment (Fig. 6). Compared to the control group, which showed diverse collagen orientation patterns, collagen became thicker and aligned horizontally, particularly evident on day-7 and day-14 following CAPJ treatment (arrowheads in the upper panels of Fig. 6). In contrast, elastic fibers thickened at day-3, with thickness gradually decreasing until day-14, while displaying a horizontal pattern akin to collagen, characterized by its time-dependent alignment (arrowheads in the middle panels of Fig. 6). Compared to the absent expression observed in the control group, hyaluronic acid exhibited heightened expression at day-3, predominantly near adipocytes, with a notable increase by day-7 (arrowheads in the lower panels of Fig. 6). These findings well align with our proteome results, demonstrating that CAPJ stimulates keratinocyte-fibroblast cross talk, leading to ECM protein synthesis and reorganization, which is central for efficient reepithelialization during the wound healing (74). This long-term influence may be linked to the elevated level of RONS induced by CAPJ, which modulates the signaling transduction and disturbs redox homeostasis during wound healing (75). Redox signaling is known to regulate the proliferation and migration of endothelium cells, keratinocytes, and fibroblasts, as well as fibroblast differentiation (76, 77). The inflammatory response, vital for successful wound closure, is modulated by RONS, which are primarily produced by neutrophils at the injury site and contribute to pathogen defense, angiogenesis, and ECM remodeling (78, 79). Consistently, our optimized CAPJ device settings (12) significantly elevated total nitric oxide (Supplemental Fig. S6*C*). Besides, the inflammation phase of wound healing was modulated, with CAPJ-treated rat wound tissues showing slightly higher expression levels of Interleukin-1β and tumor necrosis factor-alpha compared to the control group (Supplemental Fig. S6*D*). These results suggest that the controlled elevation of RONS by CAPJ not only enhances cell recruitment to the wound site but also modulates inflammatory response, thereby accelerating the healing progress.

**Fig. 7:**
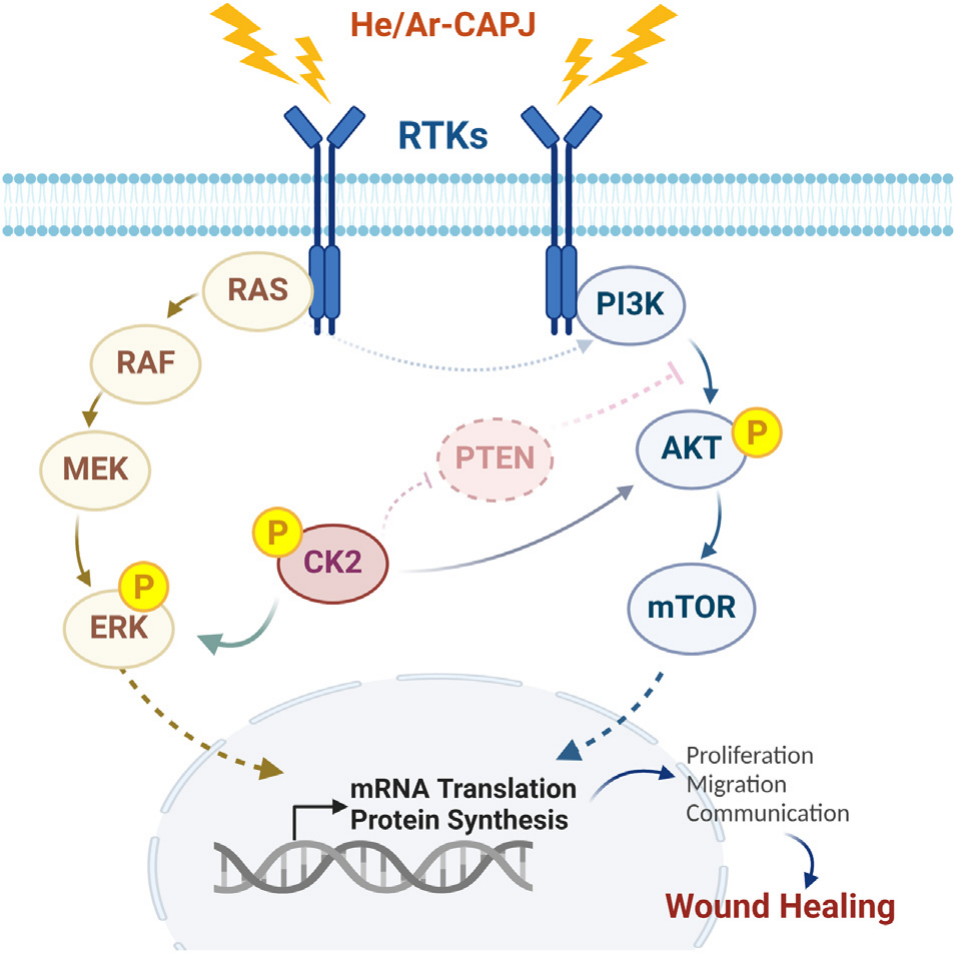
**Brief scheme of CAPJ-modulated molecular mechanisms in wound healing processes.** Proteomics analysis revealed that PAM intervention induces signal regulations involved in receptor tyrosine kinase (RTK) signaling via PI3K/AKT and MAPK pathways which promote a myriad of biological responses in wound repair to regulate cell proliferation, migration, and cell-cell communication. In particularly, CK2 functions as a multilevel modulator in the PI3K/AKT and MAPK pathways by directly phosphorylating AKT1 to promote its activity and phosphorylates PTEN to inhibit its phosphatase activity as well as assure the activation of PI3K/AKT signaling. It also phosphorylates ERK activation loop to promote its translocation into the nucleus for transcriptional regulations. These CAPJ-activated phosphosignaling events subsequently influence downstream outcomes related to keratinocyte-fibroblast cross talk and ECM remodeling during epithelization phase of wound closure. AKT, serine/threonine-protein kinase; CAPJ, cold atmospheric plasma jet; CK2, casein kinase 2; ECM, extracellular matrix; MAPK, mitogen-activated protein kinase; PAM, plasma-activated medium; PI3K, phosphoinositide 3-kinase.

In summary, CAPJ intervention activated the PI3K/AKT and MAPK signal cascades, promoting keratinocyte proliferation and migration. Also, it exerts long-term effects by stimulating keratinocyte–fibroblast cross talk and facilitating ECM remodeling, ultimately aiding in wound repair (Fig. 7).

## Discussion

CAPJ, generating a cold or nonthermal plasma comprised of various chemical active species, included RONS, represents a promising tool in biomedical applications ranging from skin wound healing and disinfection to cancer therapy (6, 10, 80). RONS serve as double-edged swords, acting as pleiotropic agents at low concentration to initiate physiologic signaling mechanisms, while higher amounts can lead to oxidative stress and cytotoxicity (76). Hence, CAPJ, as a tunable and exogeneous source of RONS, emerges to open new frontiers in plasma medicine, particularly for skin wound healing (10). Several studies have evaluated the efficacy of CAPJ in acute and chronic wound repair in animal models or patients, resulting in rapid wound closure through the coordination of cell recruitment, initiation of angiogenesis, pathogen defense, as well as immunology and inflammation modulation (12, 13, 14, 15, 16, 81, 82). However, the underlying molecular mechanisms modulated by CAPJ in wound healing are rarely investigated and need to be better characterized to utilize redox modulation as a therapeutic strategy.

In this study, we employed MS-based (phospho)proteomics to systematically dissect the effects of CAPJ on the wound healing process, utilizing keratinocyte HaCaT as a keratinocyte cell model to explore both early and prolonged molecular alterations by PAM intervention (Fig. 1*B*). Protein phosphorylation was found to undergo immediate and significant alterations after PAM treatment for 2 h, with minimal proteome changes (Supplemental Fig. S1*C*). Given the critical role of protein phosphorylation in regulating PPI formations (26, 52), our network analysis highlighted the importance of ERK, CK2, and AKT kinases in PAM-induced signal transduction (Fig. 3), with further biological validations confirming CK2 as a key coordinator in regulating the PI3K/AKT and MAPK pathways (Fig. 4). Additionally, long-term treatment of PAM indicated that CAPJ facilitated ECM remodeling during epithelization by mediating the cross talk between keratinocyte and fibroblast (Figs. 5 and 6). Concisely, these results suggest that PAM-enhanced keratinocyte migration is driven by bidirectional phosphorylation-dependent PI3K/AKT and MAPK signaling pathways, orchestrated by CK2 kinase activity, which ultimately triggers downstream physiological responses, including cell migration, cell-cell communication, and ECM turnover (Fig. 7).

Many ligand-receptor interaction pathways, such as RTKs and G protein–coupled receptor signaling, are known to respond to changes in cellular redox homeostasis, with RONS capable of directly modifying or interacting with critical molecules to regulate their activity (83). For example, both the PI3K/AKT and MAPK cascades belong to RTKs signaling, where the catalytic domains of PTEN and SHP2 can be directly oxidized by RONS to inhibit their phosphatase activity and prevent the downregulation of PI3K/AKT and MAPK signaling, respectively (77). Additionally, CK2 is important in preserving mitochondrial function, thus maintaining the redox balance to protect cells against oxidative stress and sustain cell survival signaling (84). Apparently, CAPJ-derived RONS content appropriately and effectively promoted kinase activity in PI3K/AKT and MAPK signaling pathways, fundamental mechanisms regulating many pathophysiologic processes including wound healing (85). To explore the sustained effects of CAPJ in the late phase of wound healing response, HaCaT cells treated with PAM and recovered in normal medium for an additional 24 h were collected for proteome analysis (Fig. 5). This “recovery” proteome revealed a downregulation of proteins involved in DNA repair, which could be attributed to nuclear elongation during cell migration, leading to DNA damage and disruption of DNA repair mechanisms (66). The expressions of proteins in ROBOs/SLITs signaling, as well as those involved in vesicle trafficking and fusion, especially for 14-3-3s, were significantly increased. This suggests not only increased keratinocyte mobility but also enhanced cell-cell communication *via* extracellular pathways, potentially stimulating the expression of matrix metalloproteinases in epidermal fibroblasts and promoting ECM synthesis and reorganization (59). Furthermore, we found that CAPJ effect could persist until 48 h after PAM treatment, with higher expression levels of proteins associated with cell migration and communication (Supplemental Fig. S6, *A* and *B*). Concurrently, the ECM in rat skin wound tissues treated with CAPJ underwent significant structural changes, becoming denser and more aligned, which corresponds to improved wound response and scar stiffness (Fig. 6). Overall, (phospho)proteomics approach provide valuable insights into the key cellular mechanisms and molecules involved in CAPJ-mediated wound healing. By assessing the degree of phosphorylation-dependent activation and the corresponding signaling pathways, we could refine wound classification, optimize treatment strategies, and enhance the prediction and outcomes for patients with chronic or acute cutaneous wounds treated with CAPJ.

The dysregulation of PI3K/AKT and MAPK signaling pathways have been implicated in various dermatological diseases, with focuses on hyperproliferative/hypoproliferative and inflammatory dermatoses, including wound, psoriasis, and atopic dermatitis (86, 87). The activation of PI3K/AKT and MAPK signaling is essential and can occur in different phases of wound repair, whereas aberrant signaling often impairs the injury response, leading to intensified inflammation, blood perfusion blockage, tissues necrosis, and ultimately chronic wound formation (88). In psoriasis, the hyperproliferation of keratinocyte exacerbates the progression and has been linked to the activation and phosphorylation of PI3K/AKT and MAPK signaling pathways (89). Similarly, in atopic dermatitis, an eczematous skin disorder, various cytokines contribute to its pathogenesis, with ERK kinase identified as a downstream regulator of these cytokines (90). These examples underscore the pivotal role of PI3K/AKT and MAPK signaling in the pathogenesis of cutaneous diseases and their potential as therapeutic targets to enhance skin barrier function. Furthermore, CAPJ has demonstrated clinical utility in the aforementioned cutaneous diseases in biomedical research (81, 91, 92) and has significantly improved disease severity without safety concerns. Nevertheless, not all cells or patients respond equally to CAPJ treatment. Therefore, understanding the underlying cellular mechanisms could aid in selecting suitable and reliable CAPJ treatment options for different therapeutic indications, paving the ways for personalized medicine and improving predictions and outcomes.

Due to the small sample size and low statistic power of our proteome and phosphoproteome data, we used unadjusted *p*-values (*p* < 0.05) to capture subtle biological changes in both phosphorylation sites and protein expression after CAPJ treatment, ensuring that valid, biologically meaningful changes were not overlooked by stricter statistical corrections. While we are aware that unadjusted *p*-value may increase the risk of false positives, we mitigated this by incorporating additional exploratory and confirmatory research strategies, such as FC filtering and bioinformatics analysis. Despite the lack of FDR adjustment being a limitation, key proteomic findings were validated through independent biological tests, such as drug inhibition, wound healing assays, immunoassays, and histological analysis, confirming their reliability. These results collectively suggest that CAPJ administration modestly promotes wound healing without causing substantial disruptions or unintended effects, consistent with previous studies. Additionally, this study is the first attempt to identify CAPJ’s regulatory role in wound healing, improving our understanding of therapeutic selectivity. However, given the limitations of the exploratory research model used in this study, increasing the sample size and applying more rigorous statistical analysis will be essential for the successful clinic application of CAPJ.

## Data Availability

All MS raw data and search result files that support the findings of this study have been deposited in jPOST repository (https://repository.jpostdb.org). The accession numbers are PXD051544 for ProteomeXchange and JPST003041 for jPOST.

## Supporting Information

This article contains Supporting information.

## Conflict of Interest

The authors declare no competing interests.
