# Supplementary material for: Cold Atmospheric Plasma Jet Promotes Wound Healing Through CK2-Coordinated PI3K/AKT and MAPK Signaling Pathways
Source: Mol Cell Proteomics. 2025 Apr 3;24(5):100962. doi: 10.1016/j.mcpro.2025.100962 (PMC12059340; doi:10.1016/j.mcpro.2025.100962)
Supplement: Supplemental information [file mmc1.pdf]

Supplemental Information

Cold Atmospheric Plasma Jet Promotes Wound Healing through CK2-coordinated PI3K/AKT and MAPK Signaling Pathways

Pei-Shan Wu[1,2], Tzu-Hsuan Wong[2], Chun-Wei Hou[1], Teng-Ping Chu[3,4], Jyh-Wei Lee[3,4,5,6,7], Bih-Show Lou[1,8*], Miao-Hsia Lin[2*]

Supplemental information file content

1. Figure S1

2. Figure S2

3. Figure S3

4. Figure S4

5. Figure S5

6. Figure S6

7. Supplementary Excel file. Table S1-S8 for proteome and phosphoproteome analysis of PAM-treated HaCaT cells. (Submitted as a combined Excel file).

Supplementary figures

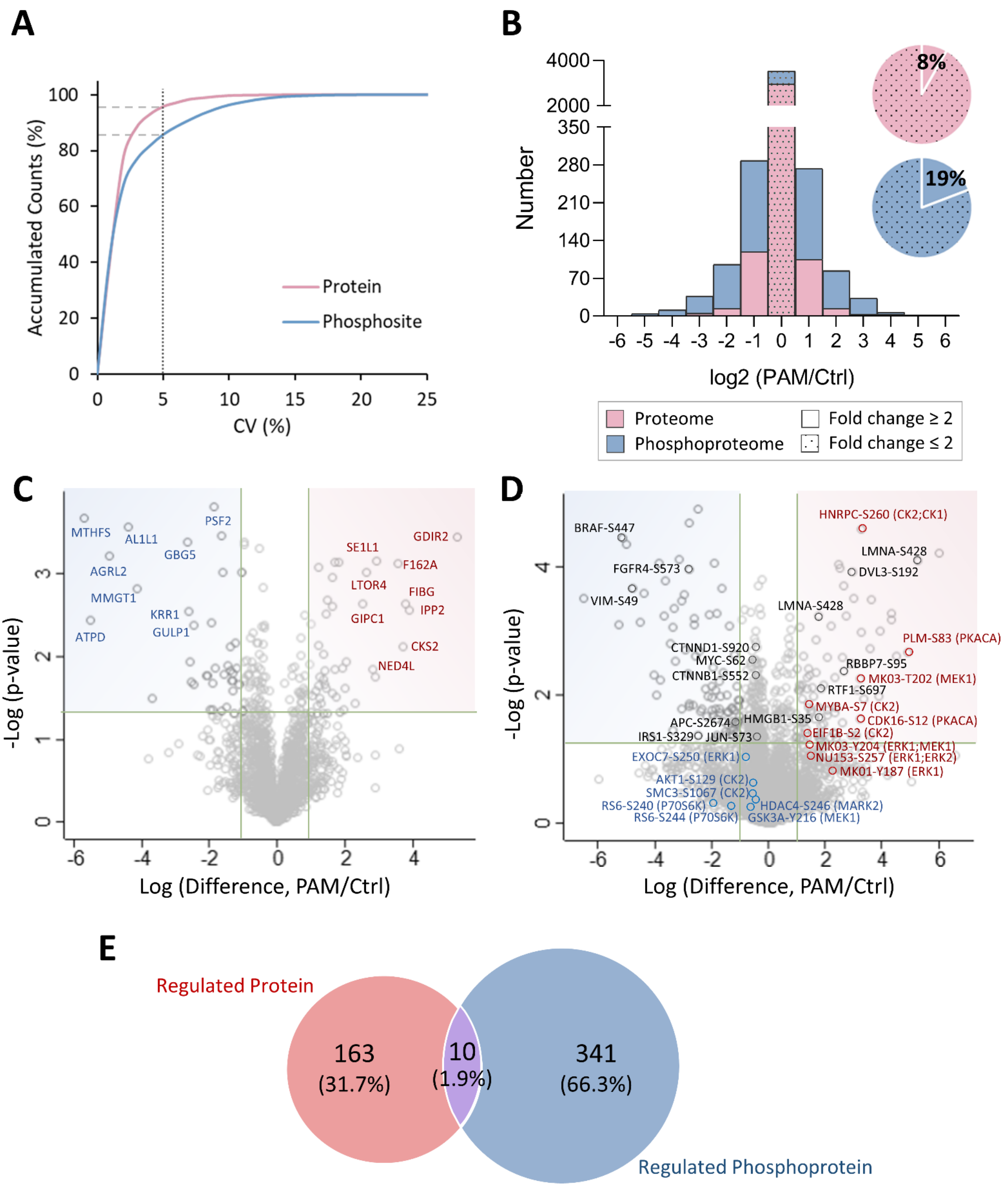

Fig. S1. Characterization of proteomics analysis in 2-hr PAM-treated HaCaT cells. (A) Comparison of coefficient of variation (CV, %) in proteome (red) and phosphoproteome (blue). Y-axis indicated the cumulative percentage of identification counts. (B) The distribution of PAM/Ctrl ratio for quantified proteins (red) and phosphosites (blue). The pie chart showed the percentage of fold-change more or less than 2-fold. Volcano plot of quantified proteins (C) and quantified phosphopeptides (D). The horizontal green-line indicated the significance at an unadjusted *p*-value of 0.05 (two sample *t*-test), and the vertical gray-lines indicated a 2-fold change difference of PAM/Ctrl. For phosphoproteome, the phosphorylated substrates and interactors of predicted kinases are highlighted in red/blue and black text, respectively. (E) Overlap of regulated proteins (red) and phosphoproteins (blue) comparing between Ctrl and PAM (two sample *t*-test without statistical correction, p-value < 0.05).

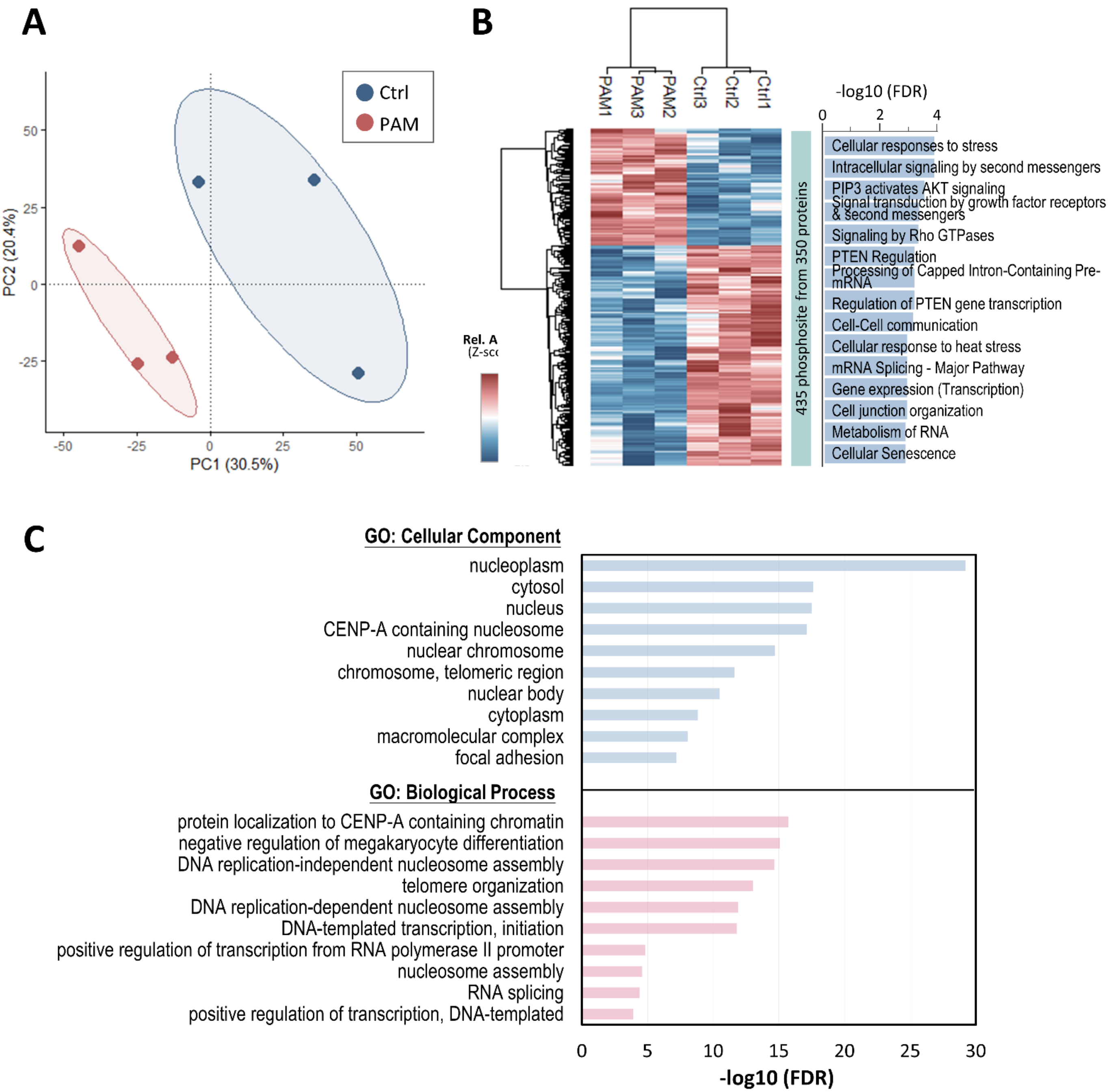

Fig. S2. Pathway enrichment and functional annotation of comparative phosphoproteome in 2-hr PAM-treated HaCaT cells. (A) PCA of 4,366 quantified class I phosphosites in the receiving phosphoproteome. Each dot represents a biological replicate and the two labels correspond to untreated (Ctrl) and PMA-treated (PAM) HaCaT cells. The 95 % confidence intervals are calculated for each group, as indicated by the blue and pink ellipses. (B) Hierarchical clustering analysis of all the altered phosphosites (left) and pathway enrichment of 350 phosphoproteins carried 435 regulated phosphosites (unadjusted *p*-value < 0.05) using Reactome pathway analysis (right bar chart). Color-indicator presents the Z-score transformed relative phosphosite abundance (Rel. Abun.). The top X-axis of bar chart is the -log10 transformed enrichment significance calculated by Benjamini–Hochberg FDR analysis. (C) GO functional annotation in terms of cellular component (blue-bar) and biological process (pink-bar) of 350 phosphoproteins using DAVID functional annotation with the significance calculated using Benjamini–Hochberg approach.

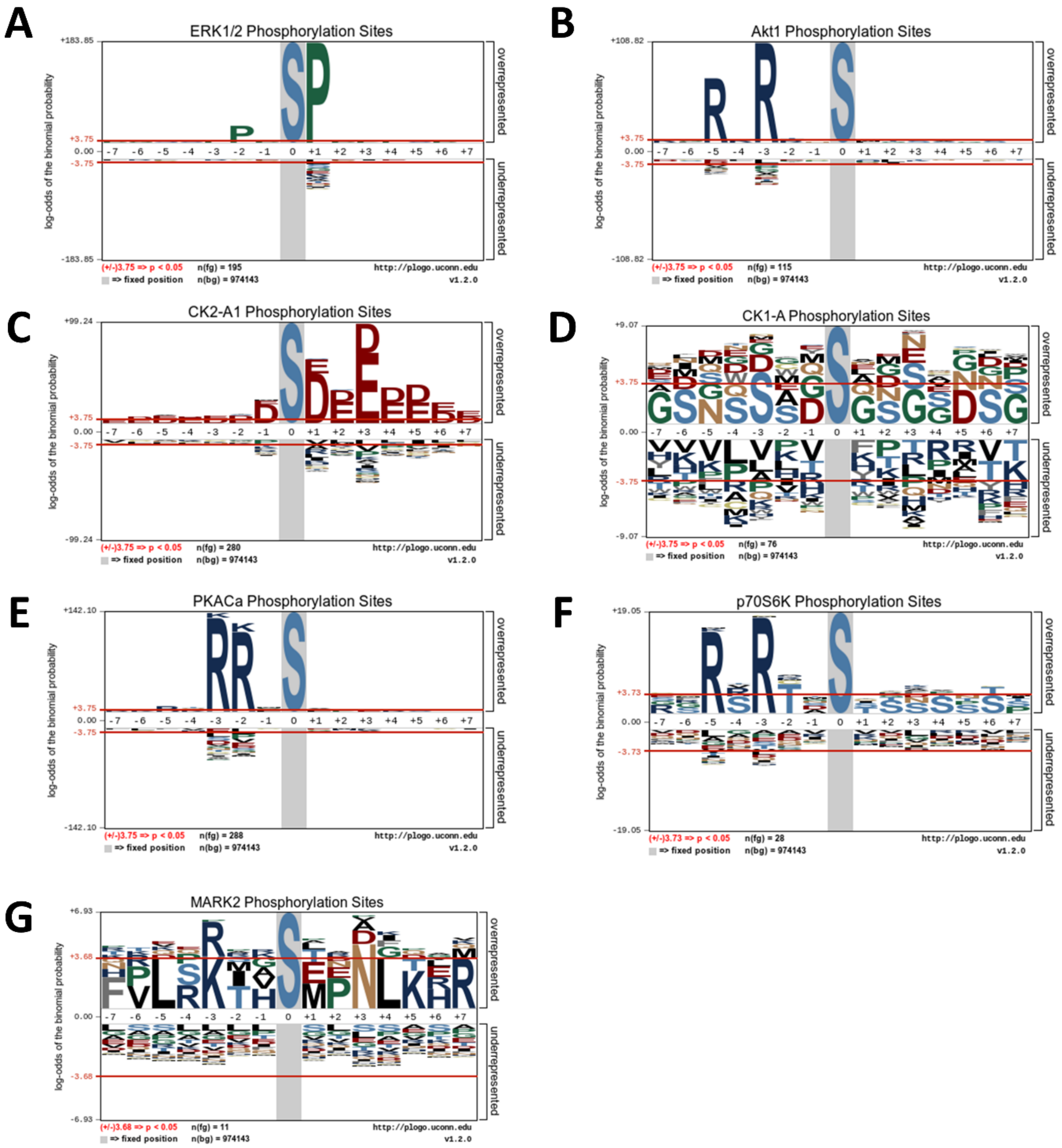

Fig. S3. Motif-logo of kinases predicted from phosphoproteome of 2-hr PAM-treated HaCaT cells. Motif-logo of PHOXTRACK predicted kinases, (A) ERK1/2, (B) AKT1, (C) CK2, (D) CK1, (E) PKACA, (F) p70S6K and (G) MARK2, were extracted by using pLogo. X-axis is the positions of the amino acid residues at C-terminal or N-terminal to the central phosphoresidue (position "0"). Y-axis is the proportional height of the amino acid residues enriched at the specific position in the pool of the queried phosphopeptides. The red horizontal bars on the pLogo correspond to p = 0.05.

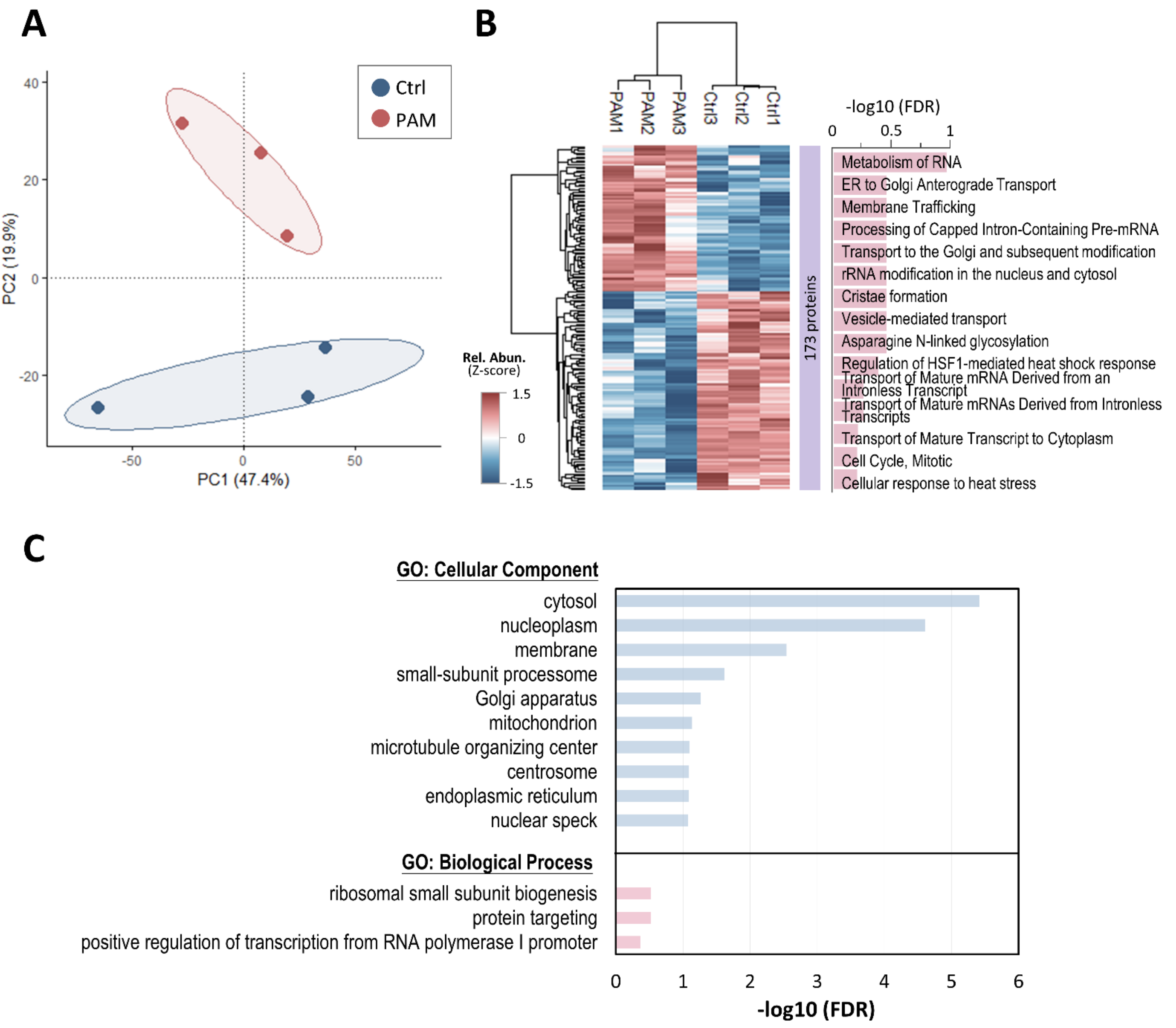

Fig. S4. Pathway enrichment and functional annotation of comparative proteome in 2-hr PAM-treated HaCaT cells (receiving proteome). (A) PCA of 3,223 quantified proteins in the receiving proteome. Each dot represents a biological replicate, with the two labels corresponding to untreated (Ctrl) and PMA-treated (PAM) HaCaT cells. The 95 % confidence intervals are calculated for each group, as indicated by the pink and blue ellipses. (B) Hierarchical clustering of 173 DEPs (unadjusted *p*-value < 0.05) along with a bar chart of the enriched Reactome pathways on the right side. The color-indicator presents the relative protein abundance (Rel. Abun.) based on the Z-score normalization. The X-axis of the bar chart is the -log10 transformed enrichment score FDR. (C) GO functional annotation in terms of cellular component (blue-bar) and biological process (pink-bar) of 173 DEPs analyzed using DAVID functional annotation tool. Both enrichment significance FDR in (B) and (C) were calculated using Benjamini–Hochberg approach.

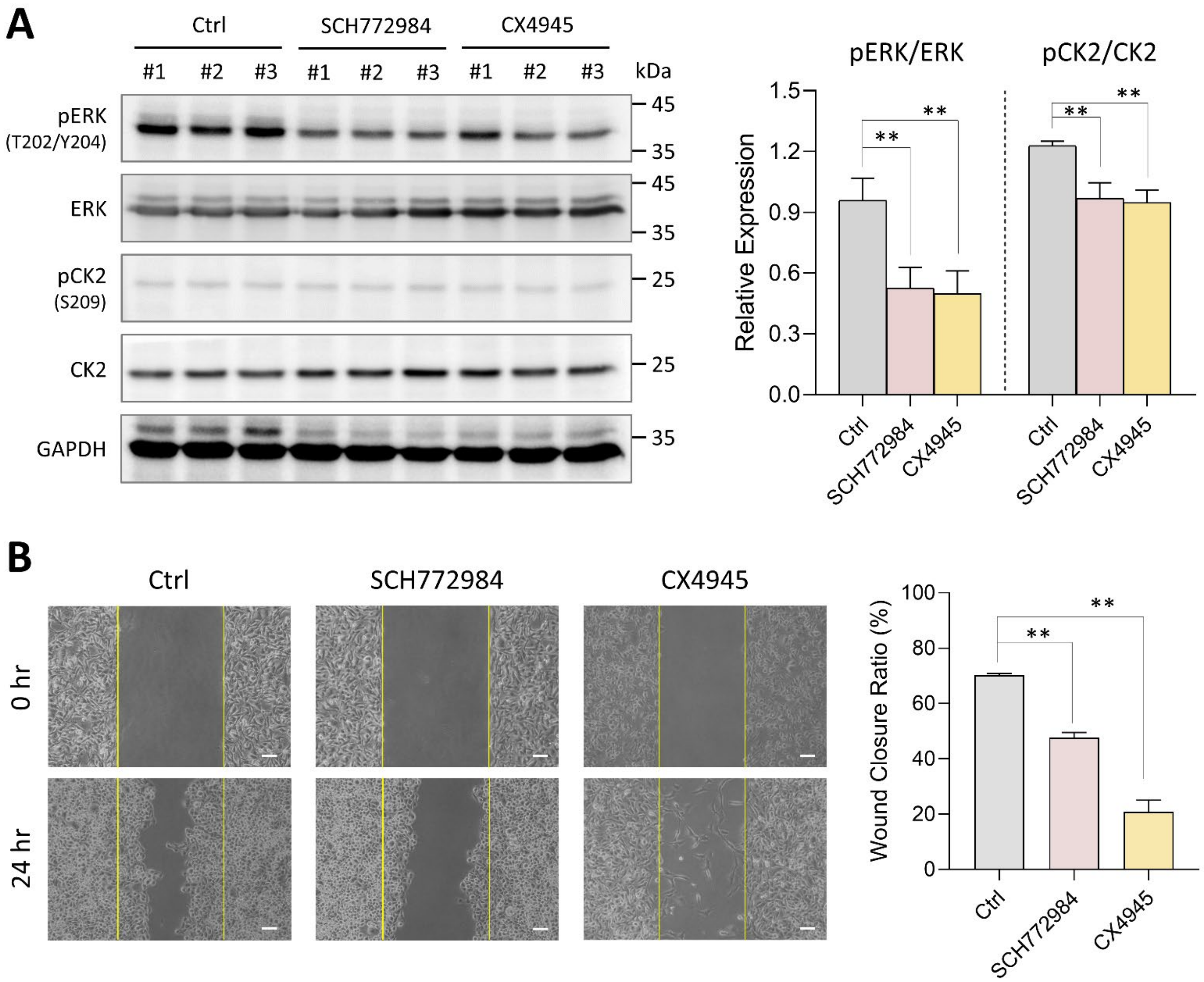

Fig. S5. Inhibition of phosphorylated ERK and CK2 in HaCaT cells. HaCaT cells are treated with ERK inhibitor (SCH772984) or CK2 inhibitor (CX4945) for 2 hours to evaluate the (A) phosphorylation changes and (B) cell migration ability. (A) Cell lysates are subjected into immunoblotting assay against indicated antibodies, accompanying with a control group (Ctrl) without any treatment. Bar charts are the densitometry analyses using ImageJ for immunoblots and present as ratio of phosphoprotein/total protein. (B) Representative images of wound healing assay in sample groups of Ctrl, SCH772984 and CX4945 for 0 hour and 24 hours incubation. Yellow dotted lines indicate the wound boundary. Scale bar= 100 µm. Bar chart illustrated the wound closure ratio (%). Data are showed as means ± SD of independent triplicate. All significance is calculated using two sample *t*-test (*p-value < 0.05, **p-value < 0.01).

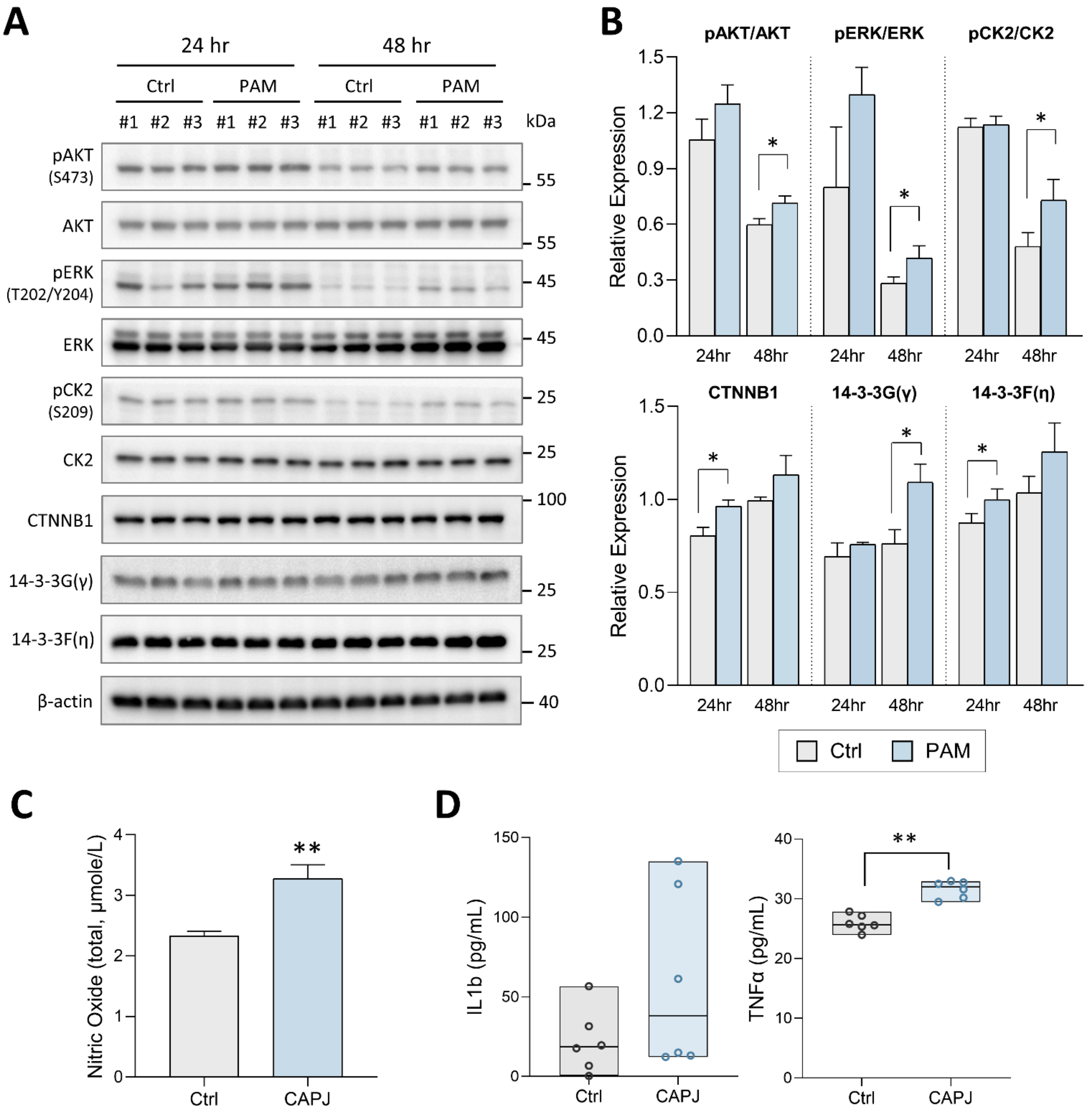

Fig. S6. Long-term effects of CAPJ treatment in wound healing process. (A) HaCaT cells treated with PAM are incubated for additional 24 and 48 hours (PAM). Cell lysates are subjected into immunoblotting assay against indicated antibodies, including pAKT, pERK, and pCK2 for signaling transduction; CTNNB1 for cell migration; 14-3-3G(γ) and 14-3-3F(η) for cell-cell communication. Cells without treatment is used as a comparative control (Ctrl) at each time point. (B) Bar charts present the quantitative densitometry of immunoblots using ImageJ (N = 3). (C) Detection of total nitric oxide detection in ddH_2O treated with and without CAPJ for 15 sec (N = 3). (D) ELISA assay for measuring IL-1β and TNF-α in rat wound tissue samples collected on Day-14. Data are presented as box plot with all points showing the concentration of IL-1β and TNFα (N = 6). All data were presented as means ± SD and the significance was calculated using two sample t-test (*p-value < 0.05, **p-value < 0.01).
